# Graphene‐Like Carbon Film Wrapped Tin (II) Sulfide Nanosheet Arrays on Porous Carbon Fibers with Enhanced Electrochemical Kinetics as High‐Performance Li and Na Ion Battery Anodes

**DOI:** 10.1002/advs.201903045

**Published:** 2020-08-20

**Authors:** Zhe Cui, Shu‐Ang He, Qian Liu, Guoqiang Guan, Wenlong Zhang, Chaoting Xu, Jinqi Zhu, Ping Feng, Junqing Hu, Rujia Zou, Meifang Zhu

**Affiliations:** ^1^ State Key Laboratory for Modification of Chemical Fibers and Polymer Materials International Joint Laboratory for Advanced Fiber and Low‐dimension Materials College of Materials Science and Engineering Donghua University Shanghai 201620 P. R. China; ^2^ College of Science Donghua University Shanghai 201620 China

**Keywords:** graphene‐like carbon, integrated carbon matrix, multi‐dimensional structures, optimized kinetics, tin sulfide arrays

## Abstract

SnS, is a promising anode material for lithium ion batteries (LIBs) and sodium ion batteries (SIBs), however, undergoes poor cyclic lifespan due to its huge volume changes and bad electroconductivity. Here, a modified CVD method is used to directly grow graphene‐like carbon film on the surface of SnS nanosheet arrays which are supported by Co‐, N‐modified porous carbon fibers (CCF@SnS@G). In the strategy, the SnS nanosheet arrays confined into the integrated carbon matrix containing porous carbon fibers and graphene‐like carbon film, perform a greatly improved electrochemical performance. In situ TEM experiments reveal that the vertical graphene‐like carbon film can not only protect the SnS nanosheet from destruction well and enhance the conductivity, but also transforms SnS nanosheet into ultrafine nanoparticles to promote the electrochemical kinetics. Systematic electrochemical investigations exhibit that the CCF@SnS@G electrode delivers a stable reversible capacity of 529 mAh g^−1^ at a high current density of 5 A g^−1^ for LIBs and 541.4 mAh g^−1^ at 2 A g^−1^ for SIBs, suggesting its good potential for anode electrodes.

## Graphical abstract

SnS nanosheet arrays sandwiched into the integrated carbon matrix containing porous carbon fibers and graphene‐like carbon film are prepared and provide a great improvement in the electrochemical performance of lithium ion and sodium ion batteries.

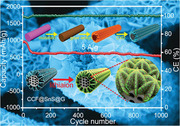

## 1 Introduction

With the rapidly increasing pursuit of portable electronic devices, large‐scale grid energy storage systems (EESs) with much higher energy density have triggered an urgent demand.^[^ ^1^ ^]^ Among various EESs such as alkali metal ion batteries, fuel cells, flow batteries, and supercapacitors, lithium ion batteries (LIBs) and sodium ion batteries (SIBs) have been acknowledged as the most promising candidates owing to their long lifespan, environmental friendliness, and no memory effect.^[^ ^2^ ^]^ However, the commercial graphite anodes neither satisfy the demand of LIBs because of its limited capacity (372 mAh g^−1^) nor apply to SIBs because of the larger Na^+^ diameter (1.02 Å).^[^ ^3^ ^]^ Thus, it is imperative to explore suitable anode materials with high performance for LIBs and SIBs.

SnS has attracted extensive attention as the most promising candidate for the anode of LIBs and SIBs, owing to its layered structure, large interlayer spacing (0.433 nm), high theoretical capacity (1137 mAh g^−1^ for LIBs and 1022 mAh g^−1^ for SIBs), and less phase transformation.^[^ ^4^ ^]^ In particular, the relatively weaker Sn—S ionic bonds and continuous lithiation mechanism (containing the initial conversion‐reaction and the subsequent alloyed reaction) are in much favor of the electrochemical kinetics and higher first‐cycle coulombic efficiency.^[^ ^5^ ^]^ In spite of these advantages, the unavoidable large volume expansion and the intrinsic poor electroconductivity of SnS during the electrochemical cycles lead to rapidly increased charge transfer resistance, electrode pulverization, and the capacity decline.^[^ ^6^ ^]^ To resolve these issues, various nanomaterials and hybrid nanocomposites have been investigated to enhance the conductivity, shorten the ion transport length, relieve the stress, and further improve the energy storage performance of SnS.^[^ ^7^ ^]^ However, the structures of SnS‐based nanomaterials collapse easily and tend to aggregate to particle clusters during the long‐term ion insertion and extraction, which would lose the advantages of nanostructures in promoting the fast electron/ion transfer among active materials.^[^ ^8^ ^]^ To take full advantage of nanostructures in improving electrochemical performance, direct growth of SnS arrays on conductive substrates is regarded as an attractive method with various merits:^[^ ^9^ ^]^ 1) each individual SnS nanosheet is anchored tightly on the conductive substrate, ensuring good electron transfer; 2) sufficient interspace among neighboring arrays can accommodate the huge volume variation and expedite quick ion diffusion and easy electrolyte infiltration so as to enable fast charging/discharging ability and good structural stability; 3) the array nanostructure with large specific surface area provides plentiful active reaction sites, which is beneficial to the electrochemical kinetics. For example, Shen et al. have reported a graphene foam backbone to grow few‐layered SnS nanosheet arrays, obtaining reversible capacity of ≈1100 mAh g^−1^ at 30 mA g^−1^.^[^ ^10^ ^]^ Nevertheless, the active materials with directly exposed surface to electrolyte usually suffer continuous pulverization during charge/discharge process, thus resulting in destroyed architecture and deteriorated electrochemical performance.^[^ ^11^ ^]^ Therefore, there is still a big challenge to exploit integrated hybrid materials of SnS arrays with stable structures and excellent electrochemical performance in LIBs and SIBs.

Recently, graphene, a blooming 2D material, has been widely investigated as a promising assistant material for LIBs and SIBs owing to its outstanding physical and chemical features such as excellent electrical conductivity, high electron mobility, robust mechanical property, and chemical stability.^[^ ^12^ ^]^ Particularly, 3D graphene‐based framework containing vertically aligned graphene nanosheets has been acknowledged as an effective strategy for enhancing conductivity, increasing active reaction sites, and effectively preventing aggregation.^[^ ^13^ ^]^ However, most approaches of constructing hierarchical graphene arrays often need complex pre‐treatment or harsh technologies.^[^ ^14^ ^]^

Herein, we present a new strategy to confine the SnS nanosheets into the Co‐, N‐modified porous carbon fibers and vertical graphene‐like carbon film for improving the electrochemical kinetics and energy storage performance. Compared with SnS/carbon fiber composites in previous reports, three advantages have been achieved in this unique structure: First, the Co‐, N‐modified carbon fiber plays the role of a conductive substrate to support SnS nanosheets arrays while the porous structure can increase the mass ratio of SnS. Second, the in situ formed graphene‐like carbon film serves as the protective shell for SnS to prevent the structure from being destructed and enhance the electronic conductivity. Third, the SnS nanosheets are compactly confined into carbon fibers and graphene‐like carbon film, which can effectively reduce the direct exposure to electrolyte and side reactions. Results demonstrated by in situ TEM experiments reveal that the integrated the graphene‐like carbon film anchored on SnS nanosheet arrays and porous carbon fibers greatly improve the electrochemical kinetics and precisely protect the unique structure of SnS nanosheet arrays, which is beneficial for improving electronic conductivity, structure robustness, capacity, and cyclic stability. As expected, the fabricated electrodes of CCF@SnS@G display a steady capacity of 529 mAh g^−1^ at 5 A g^−1^ for LIBs and 541.4 mAh g^−1^ at 2 A g^−1^ for SIBs. Furthermore, when fabricated into a full cell with LiFePO_4_ in LIBs, the electrode delivers a 120 mAh g^−1^ after 100 cycles.

## 2 Results and Discussion

**Figure 1:**
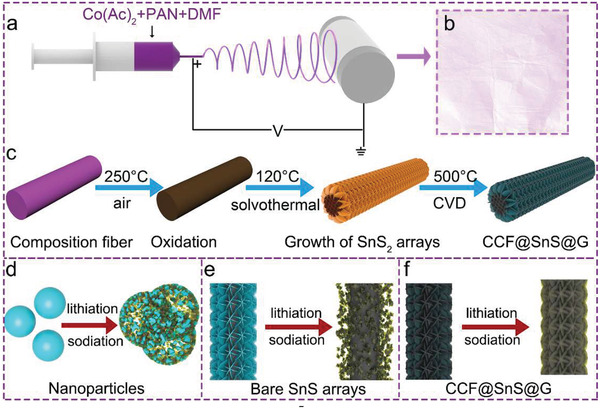
a) Schematic of the electrospinning process of PAN composited fibers. b) The photograph of composited fiber film. c) Schematic illustration of preparing CCF@SnS@G. d–f) Schematic diagrams of the structure changes for different types of nanostructures after lithiation/sodiation reaction.

The overall fabrication process for synthesizing CCF@SnS@G is illustrated in **Figure** **1**a–c. First, the polyacrylonitrile (PAN) fibers containing cobalt acetate are prepared by an electrospinning method; the fibers with a uniform morphology are in a diameter of ≈260 nm (Figure S1a,b, Supporting Information). Then the pre‐oxidation process at 250 °C in air is utilized to make the fibers stable (Figure S1c,d, Supporting Information). Subsequently, a solvothermal approach is used to grow the SnS_2_ nanosheet arrays on porous composited PAN fiber (CoPAN@SnS_2_, Figure S2, Supporting Information). Finally, the as‐prepared fibers are maintained at 500 °C under a flowing Ar/H_2_ atmosphere for a chemical vapor deposition procedure to in situ obtain the SnS nanosheets as well as interconnected graphene‐like carbon film, thus forming the final product of CCF@SnS@G. Compared with common nanoparticles (Figure 1d) or bare nanoarrays (Figure 1e), such a design has multiple advantages as shown in Figure 1f: 1) the Co‐, N‐modified porous carbon fibers and integrated graphene‐like carbon film work as a conductive framework to promote electron transport and electrochemical reaction; 2) SnS@G arrays provide enough interspace for the volume change of SnS and easy infiltration of the electrolytes; 3) the robust vertical interconnected graphene‐like carbon film serving as the protective skeleton similar to graphene can not only induce the in situ formation of SEI film layer and reduce side reactions, but also protect the structure from destruction to maintain the transport channels for electrons and ions during the cycles. In addition, the amorphous carbon coated SnS nanosheets on carbon fiber without cobalt (CF@SnS@C), the SnS arrays grown on Co, N carbon fibers (CCF@SnS) without graphene‐like carbon film, and the amorphous carbon coated SnS microflower (SnS@C) are also prepared as controllable experiments in Figures S3–S5, Supporting Information.

**Figure 2:**
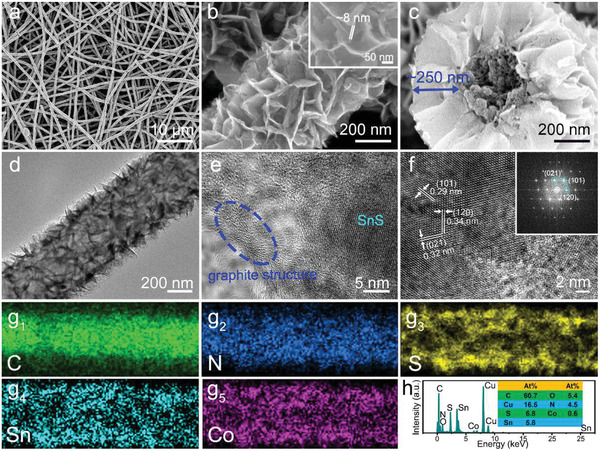
a–c) SEM images of CCF@SnS@G. d–f) TEM and HRTEM images of CCF@SnS@G. g1–g5) EDS mapping of C, N, Sn, S, Co and h) EDS spectrum of CCF@SnS@G.

The detailed morphology and structure of CCF@SnS@G are examined by scanning electron microscopy (SEM) and transmission electron microscopy (TEM) as shown in **Figure** **2**. Figure 2a shows that as‐prepared fiber‐like nanostructures inherit the integrated structures from CoPAN@SnS_2_ without any destruction, showing a uniform diameter of ≈600 nm. The densely‐packed, highly‐ordered SnS@G nanosheet arrays remain well on carbon fiber after CVD process, as shown in Figure 2b. The nanosheet arrays are interconnected and intersected with each other to form a 3D network structure with distinct gap space, which is beneficial for the penetration of electrolyte and alleviation of the volume expansion. Moreover, the ultrathin SnS@G nanosheet in a thickness of ≈8 nm could greatly increase the active sites and promote the electrochemical reactions between electrode and electrolyte.^[^ ^9c^ ^]^ The high magnification SEM image in Figure 2c shows the cross‐section of a single CCF@SnS@G fiber in which the SnS nanosheets is in a height of ≈250 nm, demonstrating abundant pores in the axial carbon fiber. These pores stemming mainly from the dissolution of Co(Ac)_2_ during the solvothermal process not only can increase specific surface area; but also are conducive to increase the mass ratio of active material in electrodes. To profoundly understand the interior structure of CCF@SnS@G, TEM images are performed. Coincided with the SEM observations above, the low‐magnification TEM image (Figure 2d) confirms the fiber‐like structure of CCF@SnS@G with the carbon fibers inside and SnS@G nanosheet on the surface. The enlarged TEM image in Figure 2e reveals that a thin graphene‐like carbon film with a thickness less than 5 nm is grown on the surface of SnS nanosheet to protect SnS nanosheets tightly. In addition, for visual observation of the integrated ultrathin graphene‐like carbon film, CCF@SnS@G was also treated by HCl solution for hours to obtain the integrated pure carbon framework shown in Figure S6, Supporting Information, which exhibits a robust 3D interconnected carbon matrix comprising of 1D porous carbon fibers and 2D graphene‐like carbon film. 5–8 layers graphite structure are observed in the inset of Figure S6b, Supporting Information. The formation of layered graphite structure should be ascribed to the catalytic activity of cobalt under reduction environment.^[^ ^15^ ^]^ These well‐aligned graphene‐like carbon film anchored closely on carbon fibers ensures the structural stability and large specific surface area as well as improves electrical conductivity, electrochemical kinetics, and accommodates the volume expansion of SnS nanosheets.

The high resolution TEM image in Figure 2f displays three obvious lattice spacing of 0.29, 0.32, and 0.34 nm, respectively corresponding to the (101), (021), and (120) crystal planes of SnS. The inset image on the upper right corner in Figure 2f is the corresponding fast Fourier‐transform pattern, confirming the well crystallized structure. The energy‐dispersive X‐ray spectroscopy (EDS) mapping is analyzed in Figure 2g1–g5 to examine the distribution of different elements in CCF@SnS@G. It clearly exhibits that C and N are mainly concentrated in the inner part, resulting from the N‐doped carbon fiber; while Sn and S are distributed on the outer sides, corresponding to the SnS nanosheets on the surface. Notably, the Co element disperses well on the whole fiber, demonstrating the uniformly existence of cobalt. Moreover, the quantitative analyses are shown in Figure 2h, from which the atom ration of Co: Sn: S is about 1:10:11, indicating the little influence of cobalt on reducing active materials due to its low content.

**Figure 3:**
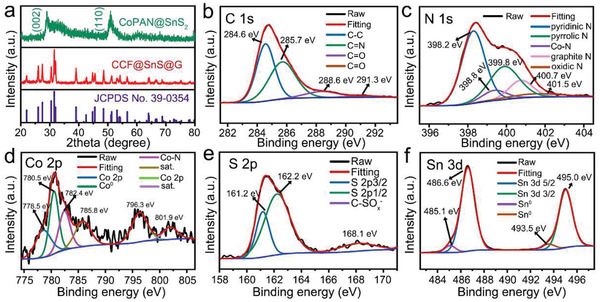
a) XRD patterns CoPAN@SnS_2_ and CCF@SnS@G. b–f) High‐resolution XPS spectra of C 1s, N 1s, Co 2p, S 2p, and Sn 3d in CCF@SnS@G.

In order to give a further comprehension for structures of the composites, X‐ray diffraction (XRD) measurement is first conducted to identify the phase compositions. As shown in **Figure** **3**a, CCF@SnS@G displays a typical XRD pattern of orthorhombic crystalline SnS without any impurity, in which all peaks can be well indexed to standard card of Joint Committee on Powder Diffraction Standards (JCPDS) No. 39‐0354. The sharp diffraction peaks suggest its higher crystallinity. The Raman spectroscopy is shown in Figure S7, Supporting Information. Two typical Raman shifts associated with the disordered graphite (D bands) at ≈1342 cm^−1^ and crystalline graphite (G bands) at ≈1571 cm^−1^ can be observed, indicating the existence of carbon.^[^ ^16^ ^]^ Compared with the CF@SnS@C and SnS@C, CCF@SnS@G has the largest value of *I*_D_/*I*_G_, implying the formation of plentiful defects due to the dual doping of Co and N atoms, which is essential for the enhancement of conductivity and active sites.^[^ ^16^ ^]^ X‐ray photoelectron spectroscopy (XPS) is utilized to confirm the valance states of CCF@SnS@G composite. Figure S8, Supporting Information, shows the feature peaks of CCF@SnS@G in the survey spectrum, confirming the presence of the elements of C, N, Co, S, and Sn. The high‐resolution C 1s spectrum in Figure 3b exhibits four subpeaks, where the two peaks at around 284.6 and 285.7 eV are respectively assigned to C—C sp2 and C=N, verifying the heteroatom doping of N in the carbon matrix.^[^ ^15^, ^17^ ^]^ The N‐doping originates from the decomposition and carbonization of both PAN and 2‐methylimidazole, and the chemical status of N could be further uncovered by the high‐resolution N 1s spectrum in Figure 3c. It is of evidence that four peaks located at 398.2, 399.8, 400.7, and 401.5 eV should belong to pyridinic N, pyrrolic N, graphitic N, and oxidized N, respectively.^[^ ^15^, ^16^ ^]^ Notably, the peak at 398.8 eV attributed to the Co—N bonding proves the existence of Co—N—C in the carbon matrix, which is commonly generated by the interaction between Co and 2‐methylimidazole.^[^ ^17^, ^18^ ^]^ The presence of Co—N*_x_* moieties is further affirmed by the analysis of high resolution Co 2p spectrum in Figure 3d, which displays the Co—N bonding at 782.4 eV;^[^ ^19^ ^]^ while the other peaks of Co 2p and Co^0^ suggest that metallic Co nanoparticles are partially presented in oxidized states, which is usually observed in Co‐containing N‐doped carbon matrix.^[^ ^15^, ^19^ ^]^ The Co and N dual doped carbon can provide higher electrical conductivity and much more activity sites for the electrochemical reactions, and thus improving the battery performance.^[^ ^17^, ^20^ ^]^ Figure 3e shows the high resolution S 2p spectrum, where the peaks could be divided into three position at 161.2, 162.2, and 168.1 eV, respectively corresponding to S 2p_3/2_, S 2p_1/2_, and sulfuric species of C—SO*_x_*.^[^ ^8^, ^21^ ^]^ The high resolution spectrum of Sn 3d in Figure 3f provides more detailed information about the chemical status of Sn. Typically, the two apparent peaks at 486.6 and 495.0 eV should be assigned to be Sn 3d_3/2_ and Sn 3d_5/2_ of Sn^2+^.^[^ ^22^ ^]^ Additionally, another two peaks of metallic Sn^0^ at 495.1 and 493.5 eV are caused by the unavoidable reduction of SnS_2_ during the carbonization process.^[^ ^7c^ ^]^ The content of SnS and carbon is explored by thermogravimetric analyses (TGA) measured under air condition (Figure S9, Supporting Information). The weight changes during the calcination process are generated from following two chemical reactions: the oxidation of carbon into CO_2_ and the transformation of SnS to SnO_2_. Therefore, the content of carbon for CCF@SnS@G and CF@SnS@C is respectively calculated to 39.6% and 59.9%. In comparison with the CF@SnS@C electrode, the CCF@SnS@G have a larger proportion of 60.4% for SnS, which is helpful for the active mass loading on the electrodes. Furthermore, the Brunauer–Emmett–Teller (BET) measurements of different products were tested and shown in Figures S10–S12, Supporting Information, CCF@SnS@G exhibits complex type H2+H3 hysteresis loops corresponding to a typical type IV isotherm, suggesting ink‐bottle pores of carbon fibers and slit shaped pores of little cracked graphite shells,^[^ ^7c^ ^]^ which is in accordance to the SEM and TEM images. Particularly, CCF@SnS@G demonstrates the largest specific surface area (101.7 m^2^ g^−1^) with most pore distributed at ≈4 nm (inset of Figure S10b, Supporting Information), which is higher than those of CF@SnS@C (24.5 m^2^ g^−1^), CCF@SnS (98.8 m^2^ g^−1^), and SnS@C (39.4 m^2^ g^−1^). The high specific surface areas with porous structure is of great importance to provide easily penetrable channels for ions and electrons in electrolytes and alleviate the huge volume changes, thereby further improving energy storage performance of the electrode.

**Figure 4:**
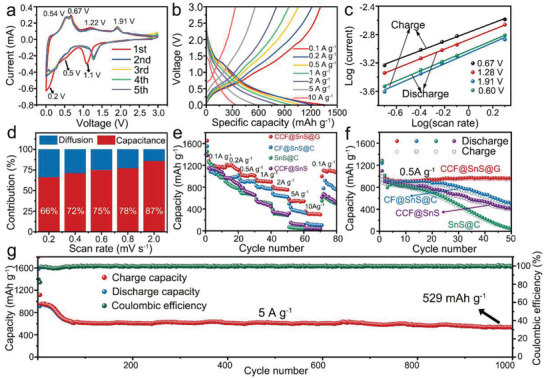
Electrochemical performance of Li^+^ storage in half cell composite. a) CV curves CCF@SnS@G at 0.1 mV s^−1^ for the initial five cycles. b) Galvanostatic charge/discharge curves of CCF@SnS@G at different current densities. c) Kinetics analysis of different peaks of CCF@SnS@G: plots of log (current) versus log (sweep rate) and the corresponding fitting line, the current and sweep rate are calculated by their absolute values. d) Contribution ratios of capacitive (red) and diffusion‐controlled contribution (blue) of CCF@SnS@G at different scan rates. e) Rate performance comparisons of CCF@SnS@G, CF@SnS@C, CCF@SnS, and SnS@C at different current densities. f) Cyclic performance comparisons of four samples at the current density of 0.5 A g^−1^. g) Long term cycles of CCF@SnS@G at 5 A g^−1^.

For the sake of validating the superiority of its unique nanostructure in energy storage performance, the electrochemical performance of CCF@SnS@G in LIB anode is evaluated in the voltage windows of 0.01–3 V (vs Li^+^/Li). **Figure** **4**a shows the cyclic voltammogram (CV) curves at 0.1 mV s^−1^ for the initial five cycles. The obvious cathodic peak at around 1.1 V in the first cycle is generated from the conversion reaction from SnS to Li_2_S and Sn (2Li + SnS → Li_2_S + Sn), while the other two little peaks at ≈0.5 V and ≈0.2 V should be ascribed to the SEI formation and the further alloyed reaction: Sn + *x*Li+ *x*e^−^ → Li*_x_*Sn (0< *x* ≤ 4.4).^[^ ^8b^ ^]^ Accordingly, two peaks at ≈0.54 V and ≈0.67 V at the anodic scan of the first cycle are attributed to the dealloying process of Li*_x_*Sn,^[^ ^8b^ ^]^ and another two peaks at around 1.22 and 1.91 V should be ascribed to the delithiation reaction from Li_2_S and Sn to SnS.^[^ ^23^ ^]^ During subsequent cycles, the overlapped peaks corresponding to the delithiation/lithiation process perform similar profiles to that of first cycle, indicating the high stability and reversibility in lithium storage reactions. It should be pointed out that the shifted peaks compared with that of the first cycle demonstrate an activation process and the irreversible formation of SEI film. Figure 4b shows the galvanostatic profiles of the CCF@SnS@G in different charge/discharge current densities. No evident plateaus in accordance to CV curves could be monitored especially at large current densities, while the voltage profile displays incremental gradients of inclination as the current continuously rises. This typical featured charge/discharge profiles of SnS‐based anode materials usually appear due to the capacitive energy storage behavior.^[^ ^7^, ^10^ ^]^ The contribution of capacitive and diffusion‐controlled capacities could be quantitatively analyzed by CV measurements employed in various scan rates (as shown in Figure S13, Supporting Information) according to Dunn's report.^[^ ^24^ ^]^ The relation between measured current (*i*) and corresponding sweep rate (*v*) can be quantified by the following equation: *i* = *a *×* v^b^*. The value of *b*, reflecting the contribution of capacitance and diffusion‐controlled capacities, could be calculated by the slope of the plot of log (*v*) versus log (*i*) in Figure 4c. The slope values of four peaks at 0.67, 1.28, 1.91, and 0.6 V are respectively 0.65, 0.68, 0.74, and 0.72, which are all between 0.5 and 1.0, indicating a mixed contribution of both two type of capacities.^[^ ^8^, ^22^ ^]^ The calculated contribution ratio is diagrammed in Figure 4d, revealing the dominating capacitive contribution of 87% at a scan rate of 2.0 mV s^−1^. In contrast, the same analyses are utilized for CF@SnS@C, CCF@SnS, and SnS@C in Figure S14, Supporting Information, demonstrating that CCF@SnS@G possesses the highest capacitance contribution ratio of all samples.

The rate performance of the four electrodes has been employed, in which the current density rises gradually from 0.1 to 10 A g^−1^ and then return to 0.1 A g^−1^. As shown in Figure 4e, the CCF@SnS@G possesses the highest capacity and consistently outperforms the other electrodes at all stages, which deliveries the average capacity of 1198.7, 1171.0, 1014.2, 906.1, 765.3, and 542.7 mAh g^−1^ respectively at the current density of 0.1, 0.2, 0.5, 1.0, 2.0, to 5.0 A g^−1^. Particularly, the CCF@SnS@G still remains the capacity of 312.2 mAh g^−1^ at 10 A g^−1^, more than 3, 7, and 12 times higher than those of CF@SnS@C, CCF@SnS, and SnS@C, respectively. Moreover, when the current is reset to 0.1 A g^−1^ again, 1045.2 mAh g^−1^ could be recovered for CCF@SnS@G, indicating a good potential of high‐power battery application. Figure 4f shows the cyclic performance of all four samples investigated at the current density of 0.5 A g^−1^. In the initial cycle, CCF@SnS@G delivers the large discharge/charge capacity of 1286.4/1037.9 mAh g^−1^, corresponding to a high coulombic efficiency of 80.6%. Subsequently, the discharge capacity is 981.4 mAh g^−1^ in the second cycle and maintains at 958 mAh g^−1^ after 50 cycles, corresponding to a capacity retention of 97.6%. Thus, the CCF@SnS@G electrode shows a decaying capacity rate of only 0.048% every cycle, which is the lowest capacity fading rate among the four electrodes (vs 0.94% of CF@SnS@C, 1.0% of CCF@SnS, and 1.9% of SnS@C). Long cycling performance at a high current density of 5 A g^−1^ has also been determined in Figure 4g. The initial five cycles are tested at a lower current density of 0.5 A g^−1^ to make the electrode activated and stabilize the structures. It shows that after the initial declining process of ≈70 cycles, 616.5 mAh g^−1^ could be obtained and a high capacity of ≈529 mAh g^−1^ is still maintained after 1000 cycles. Compared with other SnS‐based anode materials for LIBs, the CCF@SnS@G performs a good electrochemical properties (see the comparison in Table S1, Supporting Information). In particular, the capacity at high current is apparently higher than those of the SnS‐based nanostructure electrodes reported in the literatures.^[^ ^7^, ^8^, ^23^, ^25^, ^26^ ^]^

**Figure 5:**
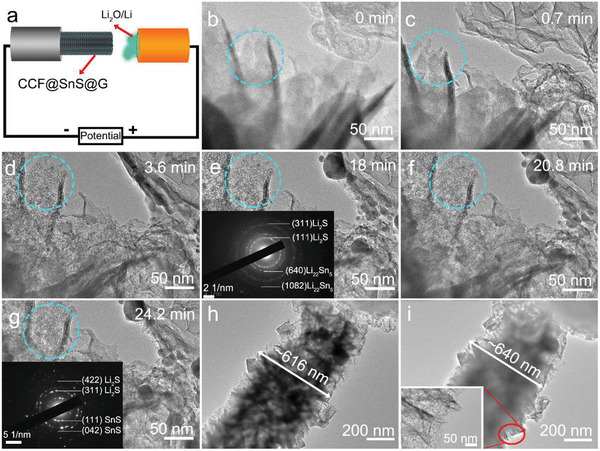
a) Schematic illustration of the in situ TEM equipment. b–g) The real time viewing of lithiation/delithiation process of SnS@G nanosheets on the surface of CCF. h,i) The morphology of a single CCF@SnS@G fiber before/after lithiation.

To further validate the as‐designed structure's advantages of CCF@SnS@G and investigate the lithiation mechanism, the real time lithiation and delithiation process of CCF@SnS@G could be monitored via an in situ TEM study.^[^ ^27^ ^]^ As shown in **Figure** **5**a, the CCF@SnS@G fiber has been anchored on the Pt probe sides as the cathode. The Au probe binding with Li/Li_2_O powders on the other side is regarded as the anode, where the Li/Li_2_O serves as both the anode material and the solid electrolyte to transfer the Li^+^ during the lithiation process. A constant potential of −3 V is utilized between the SnS@G nanosheet and the Li/Li_2_O electrode to drive lithiation reaction, as shown in Figures 5b–g. A TEM image of SnS nanosheets confined inside graphene‐like carbon film is shown in Figure 5b before lithiation process. After ≈0.7 min lithiation process, it can be observed in Figure 5c that amounts of nanopores are presented in the SnS nanosheet (blue dash circled). After ≈3.6 min (Figure 5d), the pores become clearer on the graphene‐like carbon film, and the graphene‐like carbon film displays no distinct change. After 18 min of lithiation process, no further structural change of the SnS nanosheet confined inside graphene‐like carbon film could be observed (Figure 5e), suggesting the reaction finished. Numerous ultrafine nanocrystals with a size of 3–5 nm form on the graphene‐like carbon film, which come from the SnS nanosheet after lithiation process. Moreover, the SnS nanosheet is fully lithiated into Li_22_Sn_5_ and Li_2_S, which could be confirmed by the selected area electron diffraction (SAED) pattern in the inset of Figure 5e. Furthermore, the delithiation process is also conducted by employing the opposite voltage to finish a cycle. After a continuous charging time from 20.8 min (Figure 5f), the reversible product after whole delithiation could be obtained at the time of 24.2 min (Figure 5g). The inset on the left bottom of Figure 5g confirms the existence of SnS, while the residual Li_2_S stems from the partially irreversible reaction between Li and SnS. Interestingly, the nanoparticles are still immobilized by the graphene‐like carbon film instead of fabricating into larger SnS nanoclusters or SnS nanosheet after delithiation process, which can suppress the aggregation and collapse of SnS during cycling. The unique nanocrystals of lithiated SnS and the graphene‐like carbon film can shorten the transport path of electrons and ions in lithium insertion–desertion reaction and improve conductivity and chemical reaction activity. Additionally, the single CCF@SnS@G fiber is also handled to investigate the structure and morphology changes during lithiation process. It is obviously observed that the single CCF@SnS@G fiber displays a typical expansion from ≈616 nm to ≈640 nm in diameter after lithiation (Figure 5h,i). The single fiber performs only about 4% of volume changes without structural damage, suggesting a validly stable structure for the effective alleviation of volume swelling. Moreover, from the inset TEM image in Figure 5i, the SnS@G nanosheet (red circle area) with the complete structure further manifests that there is no shedding and clustering of the arrays. Additionally, the TEM images of CCF@SnS@G after cycles in LIB clearly exhibit the entire hierarchical structure without obvious damages and cracks (Figure S15, Supporting Information), and the nanoparticles after lithiation can be observed, which is consistent with the in situ TEM observation. Thus, it has been proved by in situ TEM measurement that the robust 3D interconnected graphene‐like carbon film plays a prominent part in enhancing the electrochemical performance from at least two aspects: one is enhancing the electroconductivity and protecting the SnS nanosheet well from shedding during lithiation process; another is serving as a nanoreactor to transform the SnS into ultrafine nanoparticles without agglomeration so as to maintain the electrochemical activity.

The greatly enhanced electrochemical performance of CCF@SnS@G is also closely related with the improved charge transfer channels, which has been further studied by electrochemical impedance spectroscopy (EIS). Figure S16, Supporting Information, shows the Nyquist plots of the four electrodes after two cycles, in which the semi‐circle in high frequency area stands for the transfer resistance and the line in low frequency area represents the Warburg diffusion process.^[^ ^17^, ^25^ ^]^ It is clear that the CCF@SnS@G possesses the smallest electrical resistance, confirming its best electrical conductivity. The diffusion coefficient could be calculated by the Nyquist plots according to the formula as following: *D = R* ^2^ *T* ^2^/2*A* ^2^ *n* ^4^F^4^ *C* ^2^ *σ* ^2^, where *R* and *T* are gas constant (8.314 J mol^−1^ K^−1^) and experimental temperature (298 K), *A* is the electrode area, *n* is the number of the transfer electrons in electrochemical reaction, F is the Faraday constant (96 500 C mol^−1^), *C* is the concentration of Li^+^ in the electrode, and *σ* is the slope of the line *Z′* − *ω* ^−0.5^ respectively.^[^ ^26^ ^]^ In our experiments, the only difference among the four electrodes is the value of *σ*, which can be obtained from the line of *Z′* − *ω* ^−0.5^ (shown in Figure S17, Supporting Information). Therefore, the values of the different electrodes could be calculated to 1.0 × 10^−14^, 3.23 × 10^−15^, 1.05 × 10^−15^, and 1.04 × 10^−15^ cm^2^ s^−1^, suggesting the highest lithium diffusion coefficient of CCF@SnS@G. In addition, the EIS changes during the cycles are also investigated. In Figure S18a, Supporting Information, the CCF@SnS@G shows a continuously reduced transfer resistance and stable plots after 60 cycles, which is also the smallest and most stable one among all electrodes (as shown in Figure S18b, Supporting Information) due to the entire retention of the array structure with no breakages.

**Figure 6:**
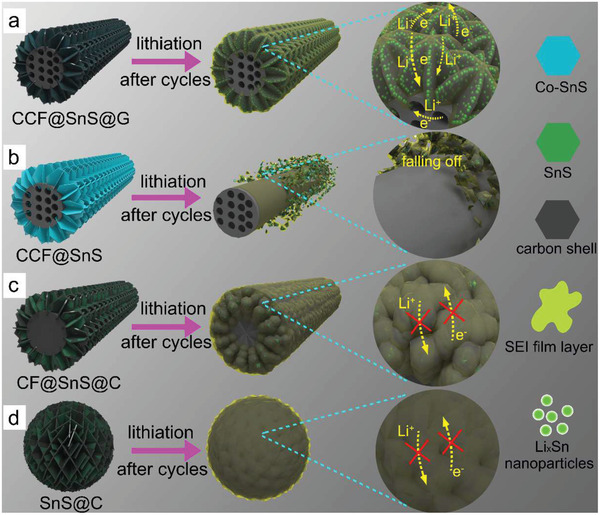
Schematic illustration of the structure changes and transfer channels of ions and electrons during the cycling process.

Based on all of analysis above, the following five strategically interdependent characteristics of the CCF@SnS@G fiber are responsible for its excellent anode performance, as illustrated schematically in **Figure** **6**a. First, the Co‐, N‐modified porous carbon fibers and robust 3D interconnected graphene‐like carbon film plays the role of a conductive substrate to ensure good electron transfer and sufficient electrochemical reactions. Second, SnS@G nanoarrays grown on CCF can provide sufficient space among neighboring array nanostructure, which can accommodate the volume change of SnS and permit easy infiltration of the electrolytes and quick diffusion of ions. Third, SnS@G nanoarrays grown on CCF can possess large specific surface area, increasing much more active sites and enhancing the capacitive contribution. Fourth, the SnS nanosheets are compactly confined between the carbon fibers and graphene‐like carbon film, which can effectively reduce the direct contact with electrolyte and side reactions as well as enhance the electrochemical activities. Finally, the vertical robust 3D interconnected graphene‐like carbon film serves as the protective skeleton for SnS to induce the SEI film to in situ form on the surface, which can prevent the structure from being destructed so as to hold the steady transport channels for electron and ion during the cycles.

Furthermore, morphologies of the CCF@SnS@G electrode before and after cycles are also examined by SEM to verify our abovementioned analysis about the outstanding anode performance. The CCF@SnS@G displays a good retention of structures without serious destruction in Figure S19, Supporting Information. On the contrary, the other three electrodes exhibit some destroyed structure, to some extent, shown in Figure S20, Supporting Information (before cycles) and Figure S21, Supporting Information (after cycles). Evidenced by these observations, it can be concluded that the bare SnS nanostructures without protective shells shed from the carbon fiber substrate during charging/discharging processes in absence of the protection of graphene‐like carbon film (Figure 6b). Furthermore, the amorphous carbon acting as protective shells for the CF@SnS@C and SnS@C leads to worse conductivity and activity, formation of thick SEI film layers, thus hindering the transport of Li^+^ and e^−^ (Figure 6c,d).

**Figure 7:**
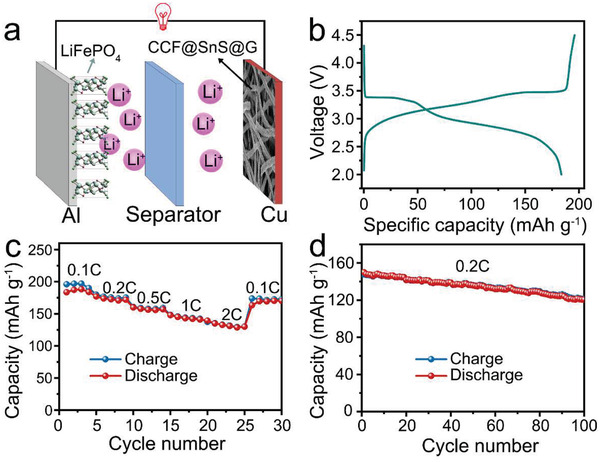
a) Schematic illustration of full LIB with commercial LiFePO_4_ powders as the cathode and CCF@SnS@G as the anode. b) Discharge/charge profiles at 0.1C. c) Rate performance of the full LIB at different current densities. d) Cycling performance of the full LIB operated at 0.2C.

To examine the electrode's potential in actual applications, the full LIBs with CCF@SnS@G as the anode and commercial LiFePO_4_ as the counter electrode are assembled as shown in **Figure** **7**a. The discharge and charge profiles in Figure 7b show two evident plateaus at ≈3.5 V and ≈3.0 V, which are respectively corresponding to lithiation reaction of SnS in the half cell. Furthermore, Figure 7c,d displays a good rate performance of ≈128 mAh g^−1^ at 2C (1C = 170 mA g^−1^, calculated by LiFePO_4_) and a reversible capacity of ≈122 mAh g^−1^ at 0.2C after 100 cycles, suggesting a great practical potential of CCF@SnS@G electrode.

**Figure 8:**
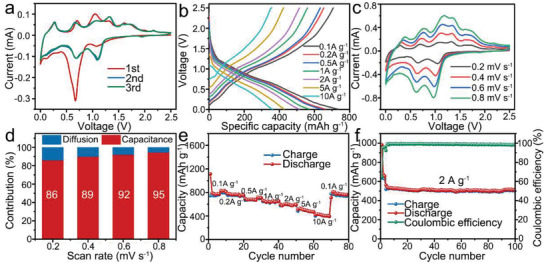
Electrochemical performance of Na^+^ storage of CCF@SnS@G as an anode in half SIB. a) CV curves of the initial three cycles. b) Galvanostatic charge/discharge profiles from 0.1 to 10 A g^−1^. c) CV curves tested from 0.2 to 0.8 mV s^−1^. d) Capacities contribution ratios of capacitive (red) and diffusion‐controlled (blue) at different scan rates. e) Rate performance comparisons at different current densities. f) Cyclic performance at the current density of 2 A g^−1^.

Furthermore, to broaden the application scope of SnS and validate its value in SIBs, the sodium storage performance of the CCF@SnS@G electrode is also evaluated by various electrochemical analysis. CV curves in **Figure** **8**a show a typical sodiation reactions between SnS and Na^+^, which is consistent with previous report.^[^ ^28^, ^29^ ^]^ The peaks at ≈1.1 V and ≈0.67 V in the initial anodic scan are respectively ascribed to the conversion reaction of SnS to Na_2_S and Sn and the alloyed reaction of Na*_x_*Sn as well as SEI film formation.^[^ ^4^, ^26^ ^]^ For the cathodic scans, the peaks at ≈0.27 V and ≈0.7 V are corresponding to the dealloying reaction of Na*_x_*Sn to Sn, while the peaks at around 1.1 and 1.3 V should be ascribed to the reversible conversion of Na_2_S and Sn into SnS phase.^[^ ^4^, ^24^ ^]^ Notably, the small weak peak at ≈1.71 V is attributed to the de‐intercalation of Na^+^ from the layered structure.^[^ ^30^ ^]^ The shift of peaks after the first cycle can be put down to the irreversible reaction with electrolytes and the formation of SEI film, and the overlapped curves of subsequent scans indicate a reasonably good stability and reversibility of the CCF@SnS@G anode. The charge/discharge profiles at different current densities are presented in Figure 8b. As the current rising, the CCF@SnS@G shows a continuously reduced capacities and imperceptible plateaus, indicating the increased proportion of capacitance contributions. CV curves at different scan rates from 0.2 to 0.8 mV s^−1^ are tested and shown in Figure 8c. The contribution of capacitance is calculated in Figure 8d, verifying a high percentage as much as 95% at 0.8 mV s^−1^. Such a high value could make great contribution to the high rate performance. As shown in Figure 8e, the CCF@SnS@G delivers ≈433 mAh g^−1^ at 10 A g^−1^, which is higher than that of LIB above because of much higher capacitive contribution. Figure 8f exhibits the cycling performance of the CCF@SnS@G at 2 A g^−1^, displaying a reversible capacity of 541.4 mA g^−1^ and a retention of 94.7% after 100 cycles. The capacity and cycling performance of the CCF@SnS@G electrode is also excellent compared with other SnS‐based materials in previous literatures (see Table S2, Supporting Information). The outstanding overall properties suggest that the CCF@SnS@G composite has a high potential as anodes for high‐performance LIBs and SIBs.

## 3 Conclusion

In summary, we have successfully designed and prepared CCF@SnS@G hierarchical structure with SnS nanosheet sandwiched into the as‐designed integrated carbon matrix consisting of porous carbon fibers and vertical graphene‐like carbon film via a simple CVD method. The in situ TEM experiments and electrochemical analyses reveal that the superior performance of CCF@SnS@G could be ascribed to the well‐designed integrated carbon matrix. The SnS@G nanoarrays grown on CCF can provide sufficient space among neighboring array nanostructure with large specific surface area and effectively reduce the direct contact with electrolyte. Besides, the Co‐, N‐modified porous carbon fibers and robust 3D interconnected graphene‐like carbon film serve as the protective skeleton for SnS to promote the SEI film layer to in situ form on the surface and prevent the structure from destruction so as to hold the steady transport channels for electron and ion. As a proof of concept, the CCF@SnS@G performs a stable capacity of 529 mAh g^−1^ for LIBs at 5 A g^−1^ and 541.4 mAh g^−1^ for SIBs at 2 A g^−1^ in cyclic performance. We believe the CCF@SnS@G is able to be a potential anode material and hope this multi‐dimensional nanostructure design be a versatile and effective strategy to build up the performance of anode materials in LIBs and SIBs.

## 4 Experimental Section

##### Synthesis of CoPAN Fibers

CoPAN fibers were obtained by an electrospinning process. The mixture solution was prepared by adding 1.0 g (CH_3_COO)_2_Co (Aladdin) and 1.0 g PAN (Macklin, *M*_w_ = 150 000) into 8 mL dimethylformamide (Sinopharm Chemical Reagent) and stirred for 12 h. The distance between needle and collector was 18 cm and the electrospun process was conducted by a constant flow of 0.1 mm min^−1^ under the voltage of 12 kV. Subsequently, the collected polymer fibers containing (CH_3_COO)_2_Co were peroxided in air at 250 °C for 2 h to obtain the Co‐PAN fibers. The pure PAN fibers were prepared under the same condition except the addition of (CH_3_COO)_2_Co.

##### Synthesis of CoPAN@SnS_2_ Arrays

The CoPAN@SnS_2_ arrays were synthesized by a solvothermal reaction. First, 1 mmol SnCl_4_·5H_2_O (Macklin) and 4 mmol thioacetamide (Sinopharm Chemical Reagent) were dissolved in 45 mL absolute ethyl alcohol and then transferred into a Teflon‐lined stainless‐steel autoclave. Then 50 mg CoPAN fibers were added into the solution and maintained at 120 °C for 9 h. After cooled down naturally, the product was washed by ethanol before dried at 60 °C overnight. The pure SnS_2_ flower was prepared by the sample process without the CoPAN fibers, and the PAN@SnS_2_ fiber was prepared under the same condition by replacing the CoPAN fibers with PAN fibers.

##### Synthesis of CCF@SnS@G

A simple chemical vapor deposition route was utilized to prepare CCF@SnS@G arrays on porous carbon fibers. Typically, two separated quartz boat respectively containing 50 mg Co‐PAN@SnS_2_ and 500 mg 2‐methylimidazole (Aladdin) were placed into a quartz tube furnace and the 2‐methylimidazole was at the upstream side. Then sample was heated to 500 °C with a ramp of 3 °C min^−1^ under a flow H_2_/Ar (10%) mixed atmosphere and maintained for 2 h. The as‐prepared SnS_2_ flower and PAN@SnS_2_ were dealt with the same process by displacing the CoPAN@SnS_2_ to obtain the controllable product SnS@C and CF@SnS@C. To obtain the pure carbon fiber without SnS (CCF@G), the CCF@SnS@G was immersed in 2 m HCl solution for 12 h and washed by water for three times before dried at 60 °C.

##### Material Characterization

The morphology of as‐prepared samples were investigated by SEM (Hitachi, S‐4800) and TEM (JEM‐2100F). XRD were conducted by a D/max‐2550 PC XRD (Rigaku, CuK*α* radiation). In situ TEM experiments were performed in TEM with the TEM‐STM holder of Nanofactory (Gothenburg, Sweden). XPS data were obtained using XPS (ESCALAB 250Xi, Thermo Scientific, USA). The Raman spectra were measured by InVia Reflex (Renishaw).

##### Electrochemical Measurements

The active material (CCF@SnS@G, CCF@SnS, CF@SnS@C, and SnS@C), conductive agent (carbon black), and binder (sodium alginate) were mixed together to prepare the working electrode in a weight ratio of 7:2:1. The mass loading ratio of active materials was 0.9–1.4 mg cm^−2^. The lithium/sodium half‐cell were fabricated into coin‐type cells (CR2032). The as‐prepared electrodes were used as working electrodes and a piece of lithium/sodium metal was used as the counter electrode. The separator was Celgard 2400 (Charlotte, NC, USA) for LIBs and glass fiber (GF/D, from Whatman) for SIBs. The electrolyte for LIBs was LiPF_6_ (1 m) in a mixture of ethylene carbonate (EC) and dimethyl carbonate (DMC) (1:1 vol%); and the electrolyte for SIBs was 1 m NaClO_4_ in in a mixture of EC and DMC (1:1 vol%) with 5.0% fluoroethylene carbonate (FEC). For preparing the lithium full battery, LiFePO_4_ was used as the cathode and the mass loading was calculated according to the capacity ratio (about 1:1.1) between cathodes and anodes. All electrochemical measurements were tested at a voltage window of 0.01–3.0 V for LIBs and 0.01–2.5 V for SIBs. The galvanostatic measurements were carried out on an LAND battery tester (CT2001A) at various current rates. CV measurements and EIS data in the frequency of 0.01 Hz to 100 kHz were conducted on an Autolab electrochemical workstation (PGSTAT302N potentiostat).

## Conflict of Interest

The authors declare no conflict of interest.

## Supporting information

Supporting Information
