# Supplementary material for: Graphene‐Like Carbon Film Wrapped Tin (II) Sulfide Nanosheet Arrays on Porous Carbon Fibers with Enhanced Electrochemical Kinetics as High‐Performance Li and Na Ion Battery Anodes
Source: Adv Sci (Weinh). 2020 Aug 20;7(18):1903045. doi: 10.1002/advs.201903045 (PMC7509643; doi:10.1002/advs.201903045)
Supplement: Supplementary file 1 — Supporting Information [file ADVS-7-1903045-s001.pdf]

© 2020 Wiley-VCH GmbH

ADVANCED SCIENCE
Open Access

Supporting Information

for *Adv. Sci.*, DOI: 10.1002/advs.201903045

Graphene-Like Carbon Film Wrapped Tin (II) Sulfide Nanosheet Arrays on Porous Carbon Fibers with Enhanced Electrochemical Kinetics as High-Performance Li and Na Ion Battery Anodes

Z. Cui, S.-A. He, Q. Liu, G. Guan, W. Zhang, C. Xu, J. Zhu, P. Feng, J. Hu,* R. Zou,* M. Zhu*

Copyright WILEY-VCH Verlag GmbH & Co. KGaA, 69469 Weinheim, Germany, 2020.

Supporting Information

Graphene-like Carbon Film Wrapped Tin (II) Sulfide Nanosheet Arrays on Porous Carbon Fibers with Enhanced Electrochemical Kinetics as High-Performance Li and Na Ion Battery Anodes

Zhe Cui, Shu-Ang He, Qian Liu, Guoqiang Guan, Wenlong Zhang, Chaoting Xu, Jinqi Zhu, Ping Feng, Junqing Hu*, Rujia Zou* and Meifang Zhu*

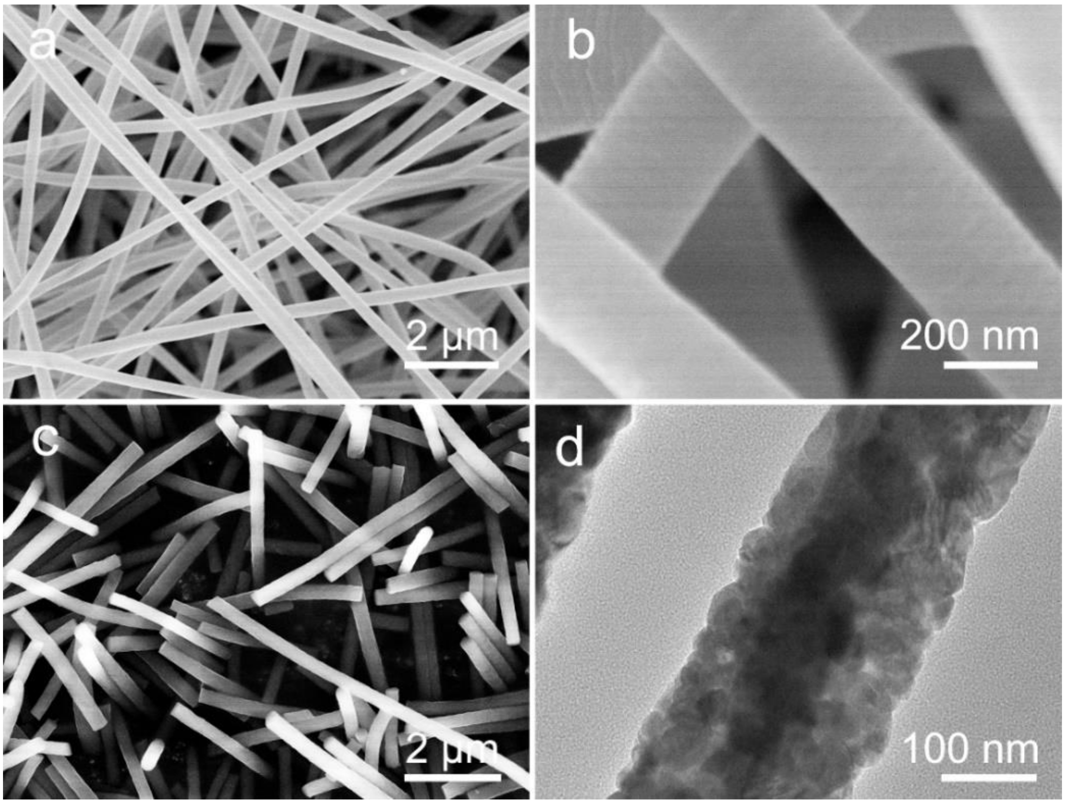

Figure S1 The SEM images of compostied fibers of $Co(Ac)_2$ and PAN (a,b) and the SEM and TEM images (c, d) of the oxidized fibers.

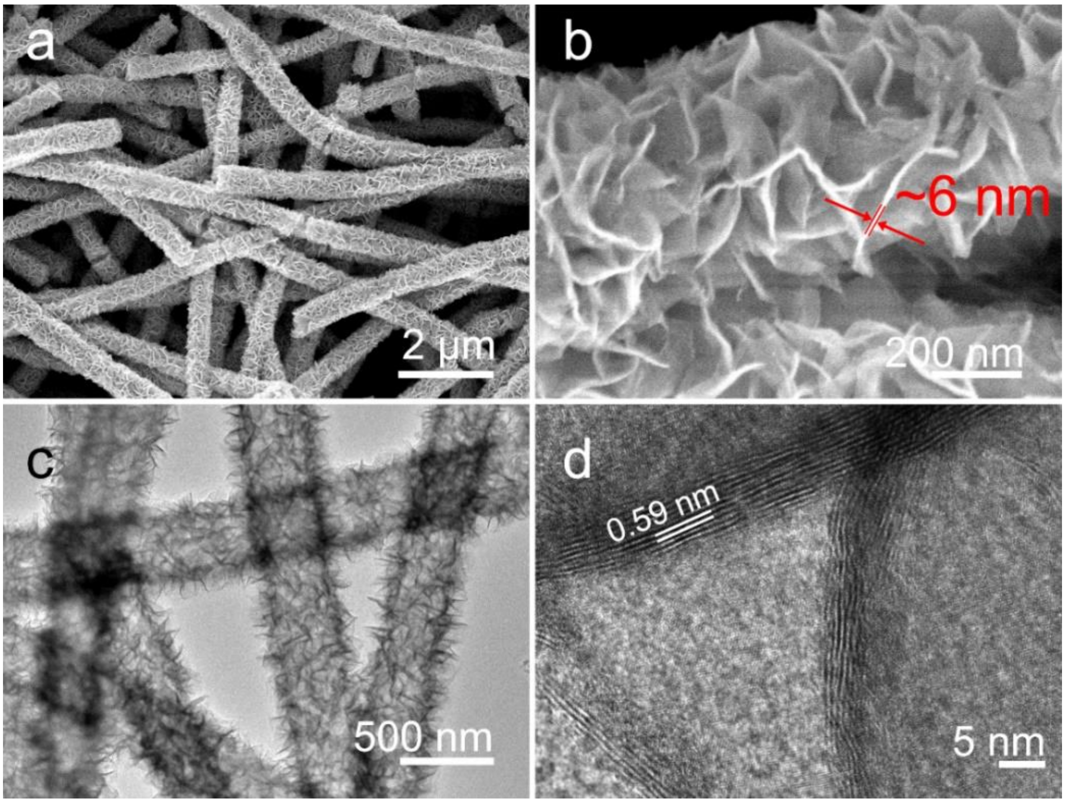

Figure S2 The SEM (a,b) and TEM (c,d) images of CoPAN@SnS_2. The ultrathin SnS_2 nanoflakes in a thickness less than 10 nm grow uniformly on the surface of fibers.

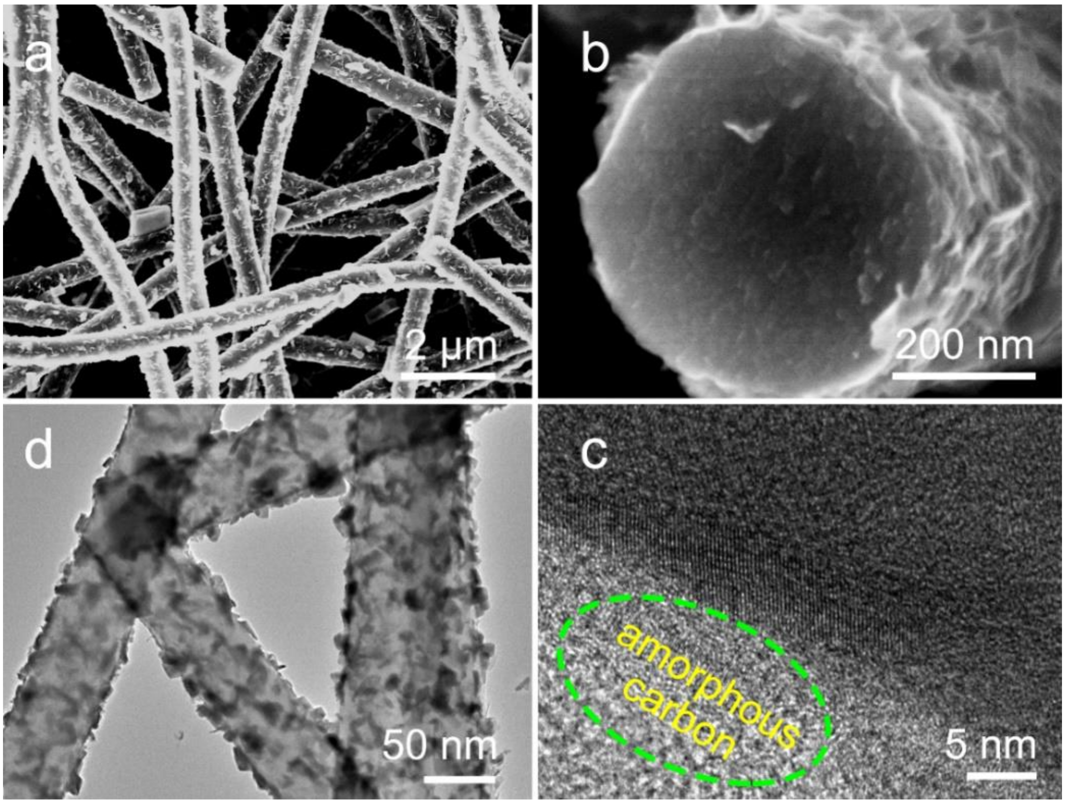

Figure S3 The SEM images (a,b) and TEM images (c,d) of CF@SnS@C, which is prepared by a similar process to that of CCF@SnS@G without adding Co(Ac)$_2$ in the electrospinning process. The CF@SnS@C contains uneven SnS nanosheets and the amorphous carbon layer on the surface as well as the solid carbon fiber inside, which could infer two points: The existence of cobalt is essential for the graphitization of carbon, and it is also beneficial for growing the more regular SnS nanosheets on the surface of PAN fibers.

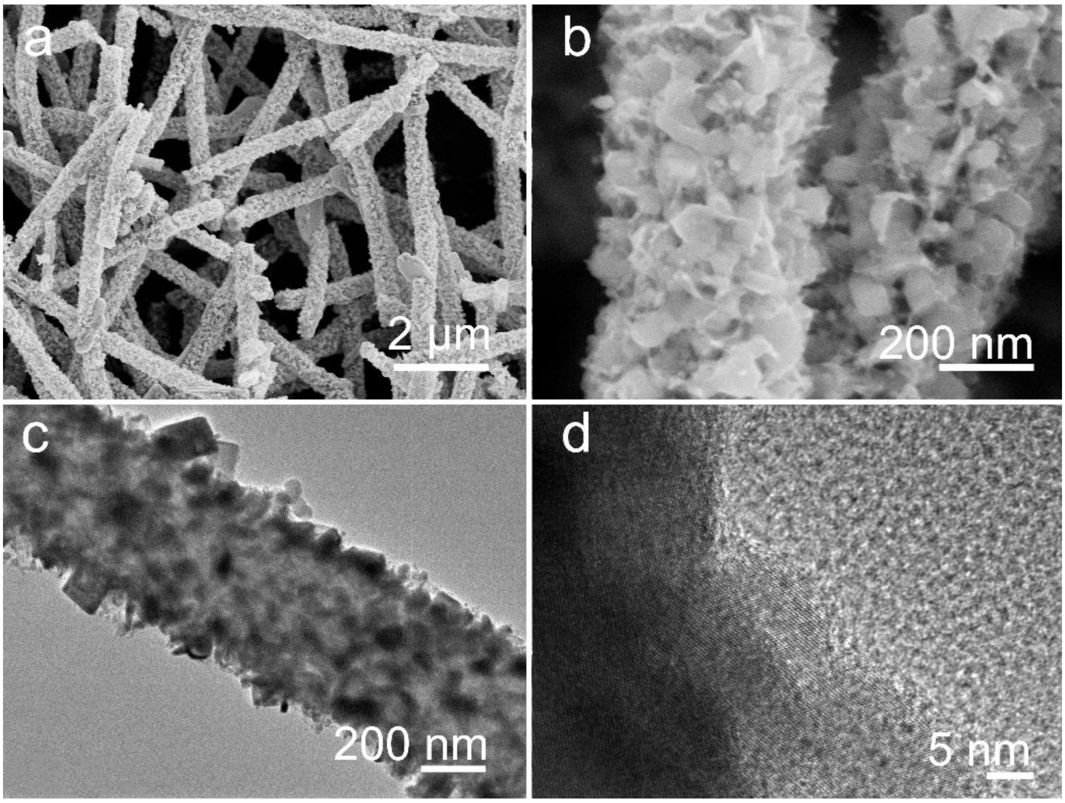

Figure S4 The SEM images (a,b) and TEM images (c,d) of CCF@SnS, which is synthesized by the same procedure with that of CCF@SnS@G, in the absence of 2-Methylimidazole during CVD process. There are obviously plentiful of large and thick nanoplates and nanoparticles on the surface of fibers, Compared with the morphology of CCF@SnS@G, it could be concluded that the 2-Methylimidazole plays an important role in maintaining the original structures of ultrathin SnS$_2$ nanosheets as well as forming graphene-like carbon film.

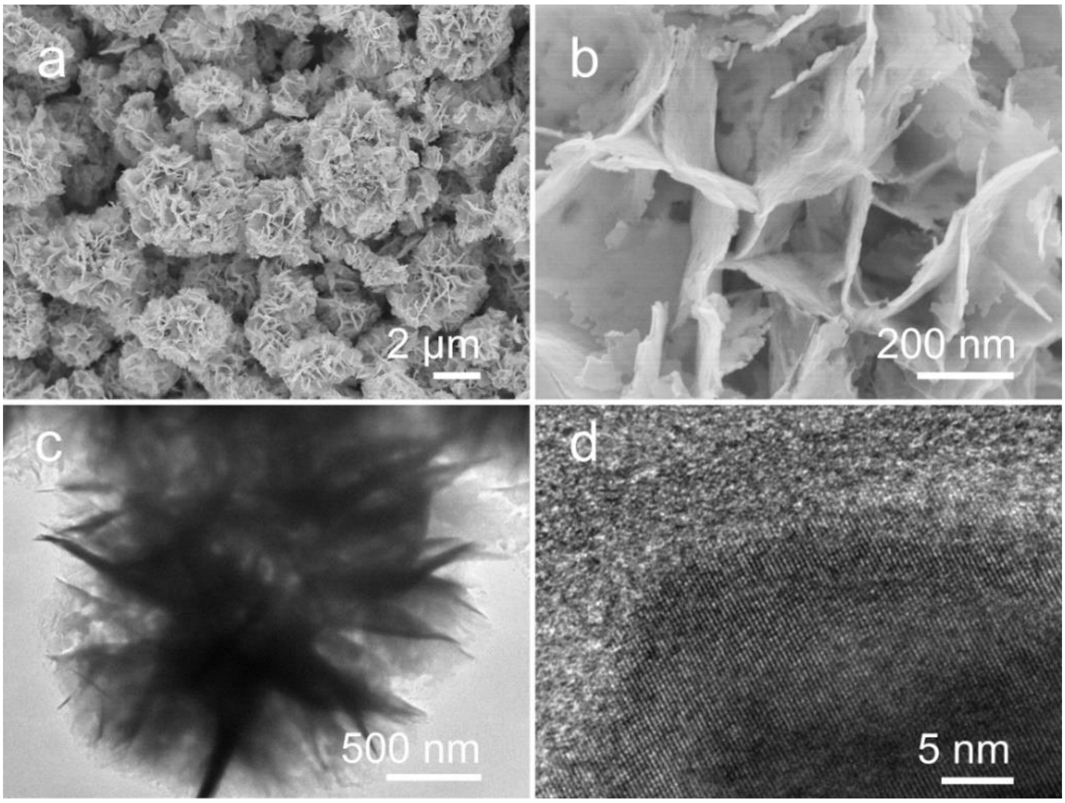

Figure S5 The SEM images (a,b) and TEM images (c,d) of SnS@C, which is fabricated by a direct solvothermal process to obtain SnS_2 first and a CVD process later to coat a carbon l ayer. The SnS nanosheets inherit the hierarchical flower-like structure from SnS_2 and are coated by a layer of amorphous carbon shell.

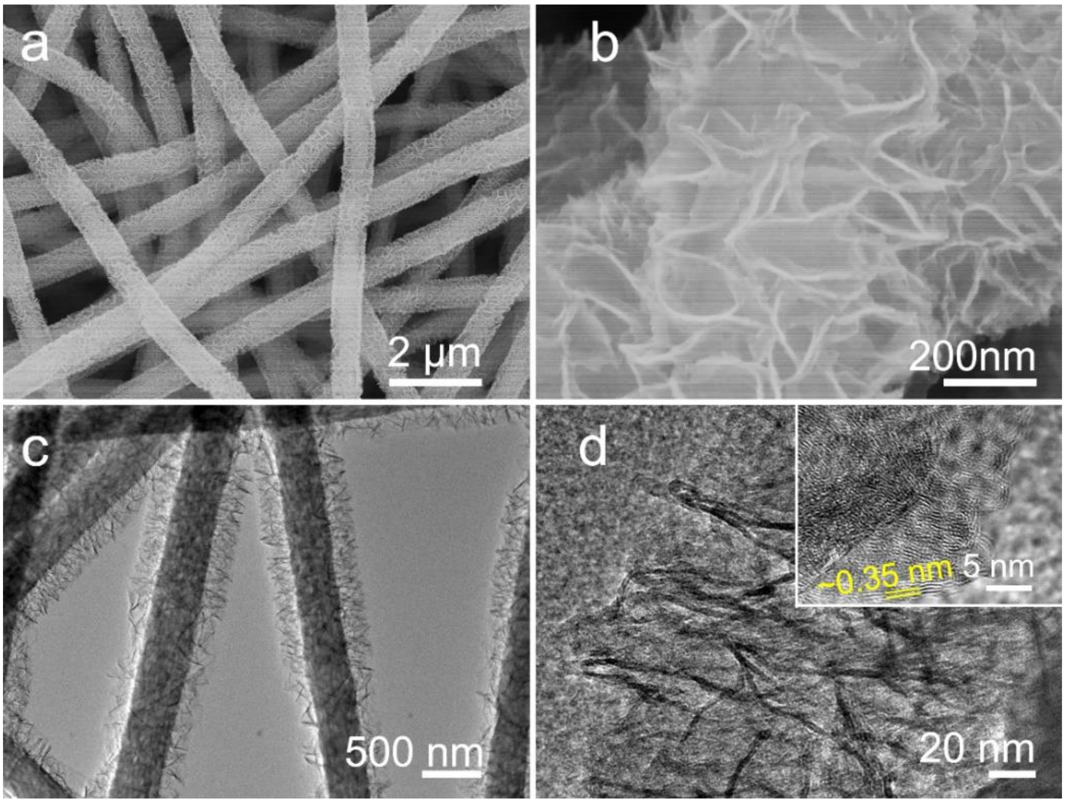

Figure S6 The morphologies of integrated carbon matrix prepared by dissolve the CCF@SnS@G using 2 M HCl solution for 12 h: (a,b) SEM images and (c,d) TEM images. It can be seen that the graphene-like carbon film grow directly on the cabon fibers and the HRTEM in the inset of Figure S3d reveals the layerd structures.

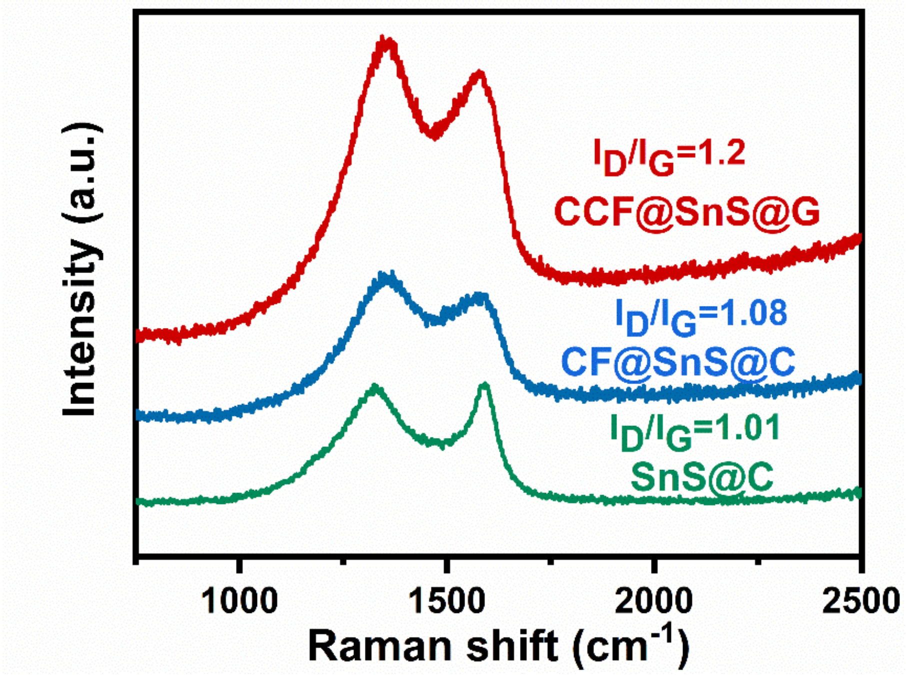

Figure S7 Raman spectroscopy of CCF@SnS@G, CF@SnS@C and SnS@C.

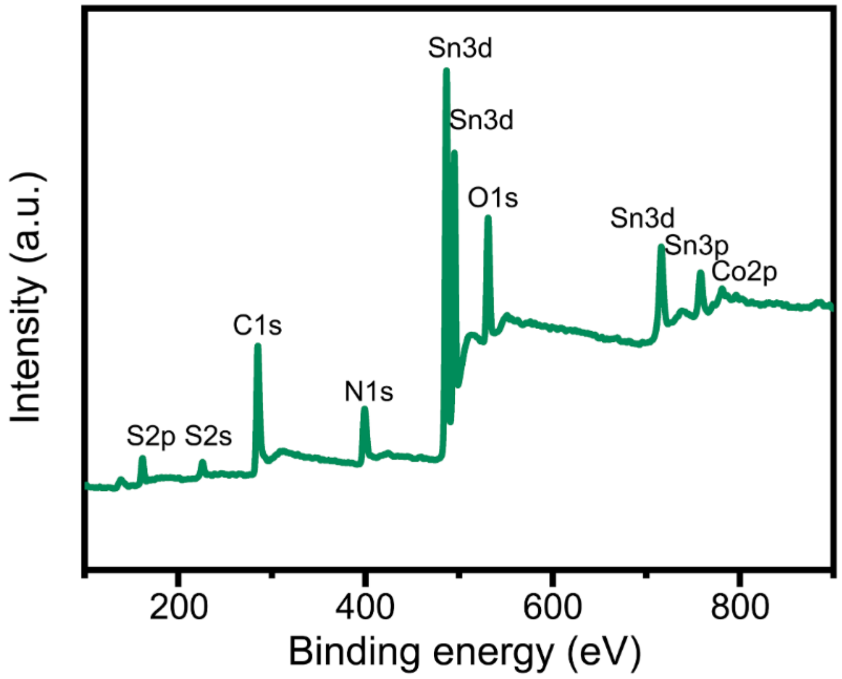

Figure S8 The XPS survery spectrum of CCF@SnS@G.

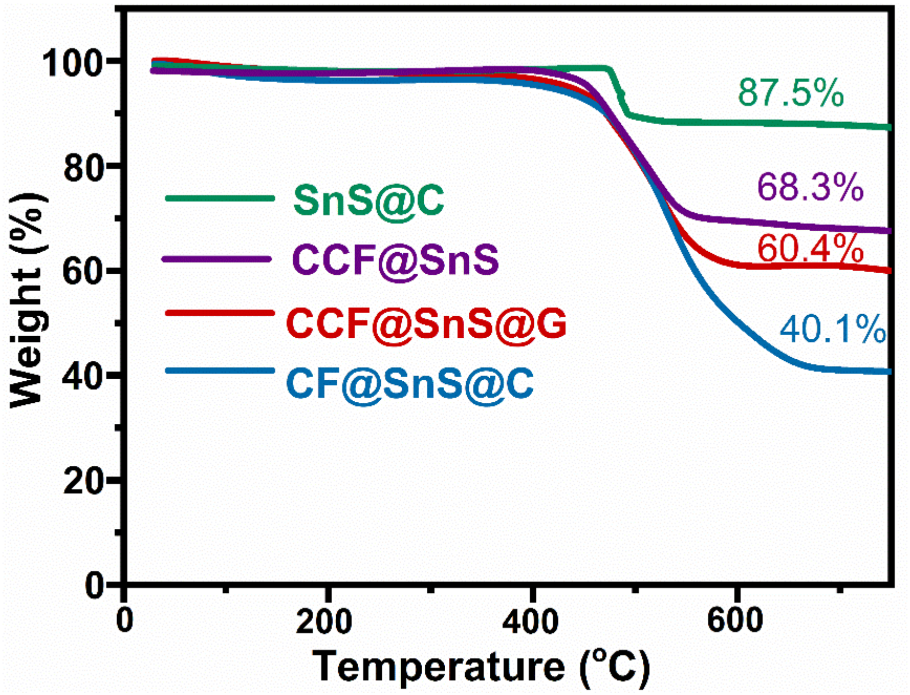

Figure S9 The TG curves of different products.

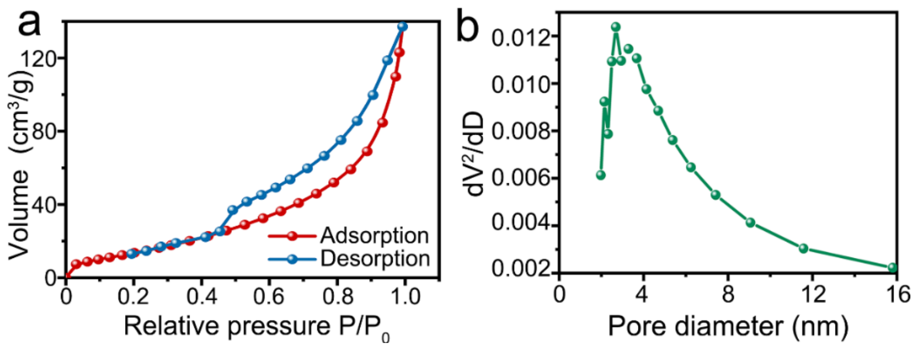

Figure S10 BET isotherm plots and corresponding BJH pore size distributions (inset) of CCF@SnS@G.

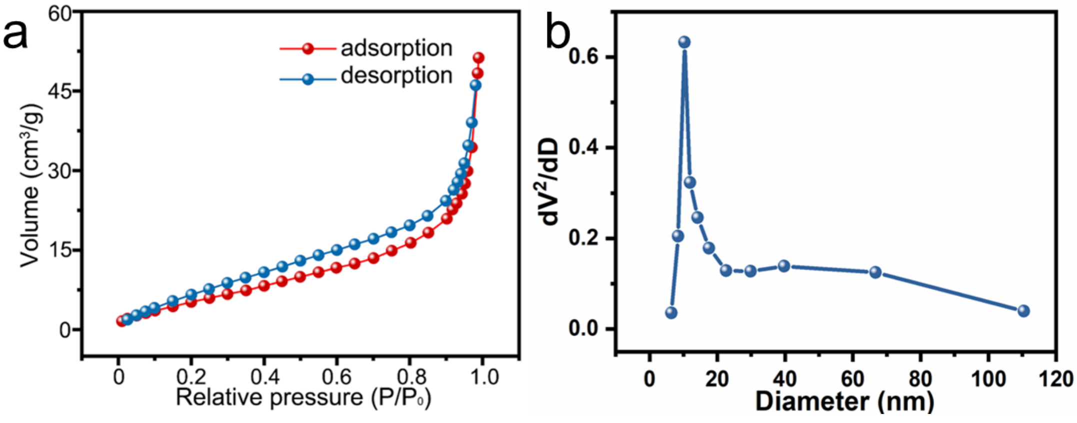

Figure S11 BET isotherm plots (a) and corresponding BJH pore size distributions (b) of CF@SnS@C.

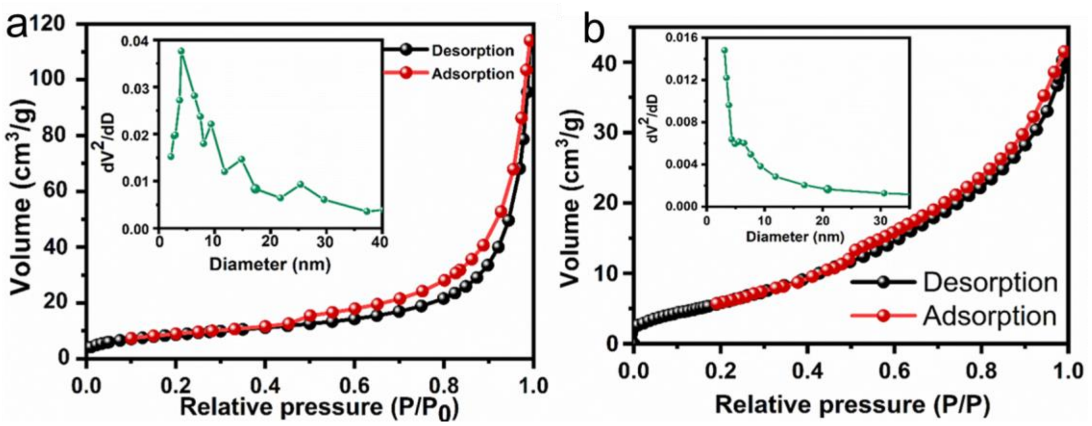

Figure S12 BET isotherm plots and corresponding BJH pore size distributions (inset) of (a) CCF@SnS and (b) SnS@C.

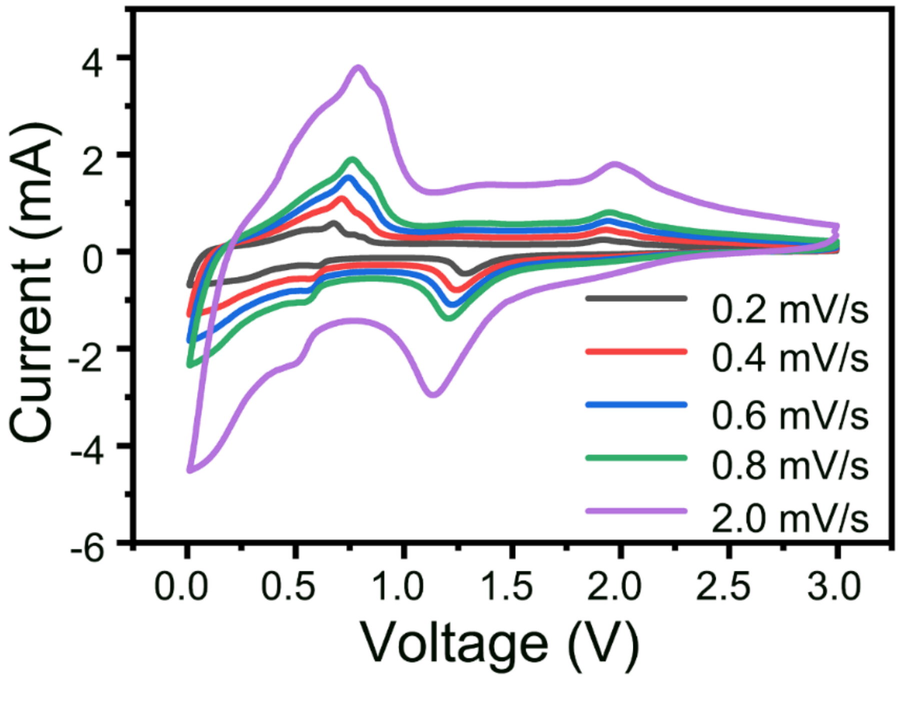

Figure S13 CV curves of CCF@SnS@G at different scan rates. The scan-rate-dependent CV curves are usually used to quantify the contribution from capacitive effects (both surface pseudocapacitance and double-layer capacitance) and diffusion-controlled Li^+ insertion process to the current response according to the following equation:

$$\boldsymbol{I\ (V) = k_1 v + k_2 v^{1/2}} \qquad \textbf{(1)}$$

where $\boldsymbol{I\ (V)}$, $\boldsymbol{k_1 v}$ and $\boldsymbol{k_2 v^{1/2}}$ represent the total current response at a given potential *V*, current due to surface capacitive effects, and current due to diffusion-controlled Li^+ insertion process, respectively. The above equation can also be reformulated as:

$$\boldsymbol{I\ (V)/\ v^{1/2} = k_1 v^{1/2} + k_2} \qquad \textbf{(2)}$$

By plotting $\boldsymbol{I\ (V)/\ v^{1/2}}$ vs. $\boldsymbol{v^{1/2}}$ at different potentials, the values of $\boldsymbol{k_1}$ (slope) and $\boldsymbol{k_2}$ (intercept) can be calculated from the straight lines, thus obtaining the contribution of capacitive effects by icaculating the integral of $\boldsymbol{k_1 v}$ with respect to a potential.

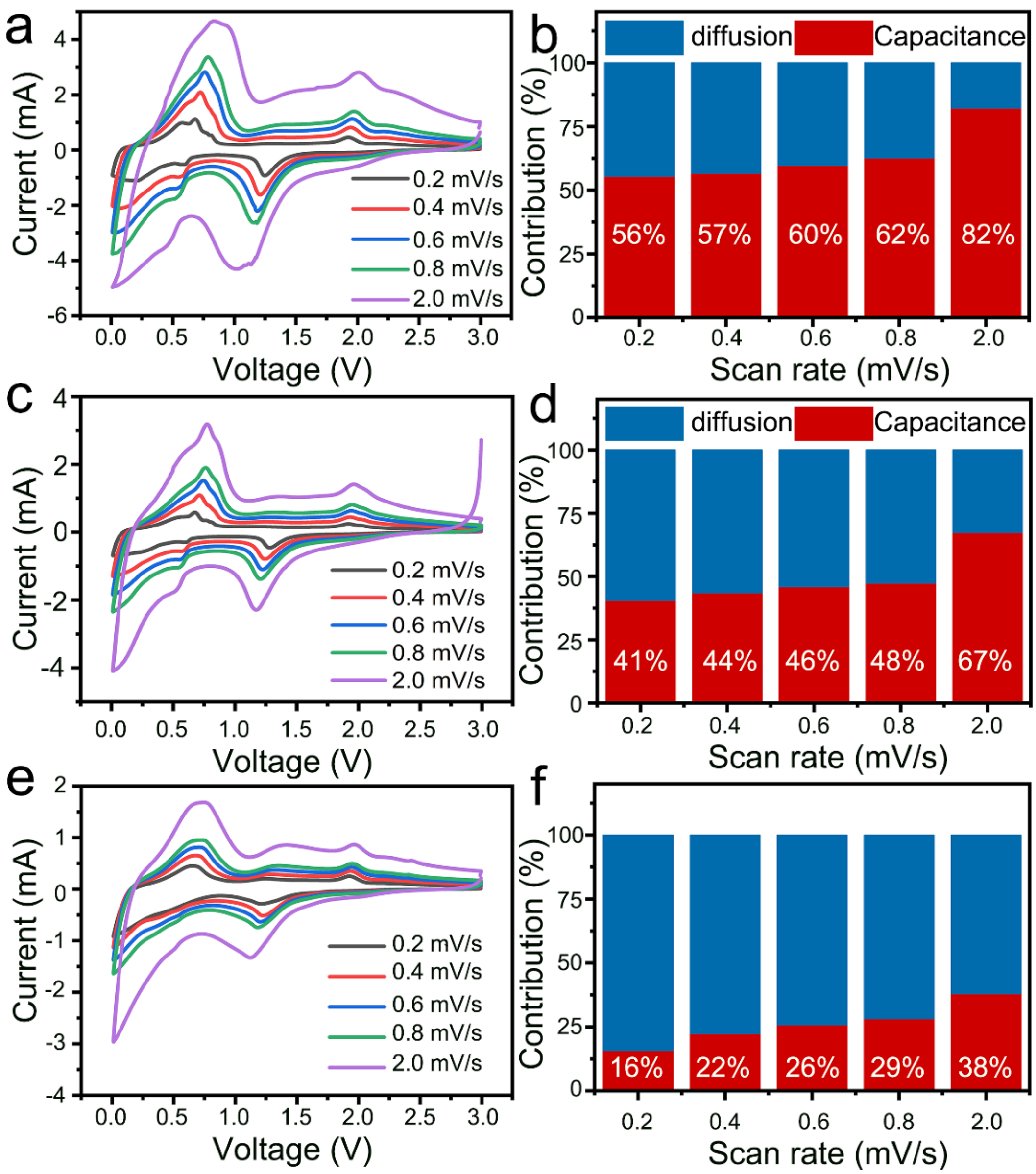

Figure S14 CV curves and the capacitance contribution ratio of CF@SnS@C (a,b), CCF@SnS (c,d) and SnS@C (e,f).

Table S1 The electrochemical performance of SnS-based materials in LIBs

Materials	High rate	Cycle performance	References
SnS@C-rGO	230 mAh g^{-1}@3.2 A g^{-1}	1027 mAh g^{-1}@0.2 A g^{-1}, 100 cycles	1

3D porous interconnected SnS/C	329 mAh g^{-1}@10 A g^{-1}	607 mAh g^{-1}@1 A g^{-1}, 200 cycles	2
SnS/C nanofiber	206 mAh g^{-1}@4 A g^{-1}	330 mAh g^{-1}@0.8 A g^{-1}, 1000 cycles	3
SnS/N-RGO	~300 mAh g^{-1}@2 A g^{-1}	229 mAh g^{-1}@2 A g^{-1}, 200 cycles	4
Coconut-Like SnS/C Nanospheres	557 mAh g^{-1}@5 A g^{-1}	830 mAh g^{-1}@0.5 A g^{-1}, 250 cycles	5
NG-SnS	378 mAh g^{-1}@5 A g^{-1}	790 mAh g^{-1}@0.5 A g^{-1}, 900 cycles	6
SnS/N-G	340 mAh g^{-1}@3 A g^{-1}	758 mAh g^{-1}@0.5 A g^{-1}, 400 cycles	7
SnS/PDDA-Ti_3C_2	471 mAh g^{-1}@2 A g^{-1}	681 mAh g^{-1}@0.5 A g^{-1}, 100 cycles	8
3D SnS/C	550 mAh g^{-1}@3 A g^{-1}	869 mAh g^{-1}@1 A g^{-1}, 1000 cycles	9
CCF@SnS@G	312.2mAh/g@10 A g^{-1}	529 mAh g^{-1}@5 A g^{-1}, 1000 cycles	**This work**

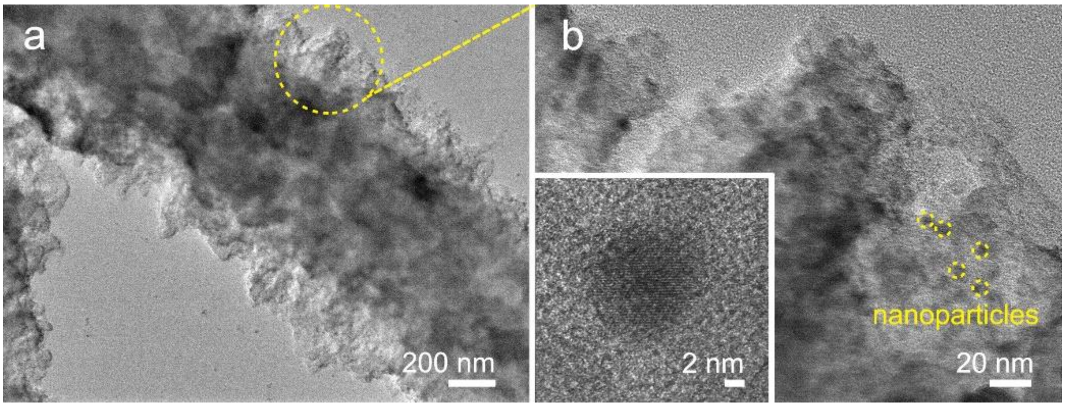

Figure S15 The TEM images of CCF@SnS@G after cyles.

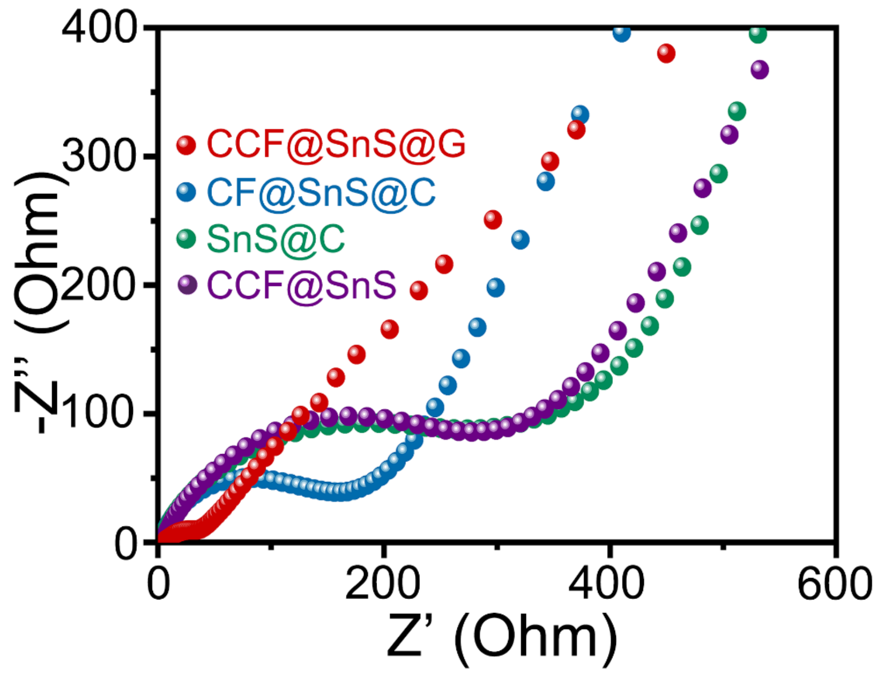

Figure S16 Nyqiust plots of four electrodes of CCF@SnS@G, CF@SnS@C, SnS@C and CCF@SnS after two cycles.

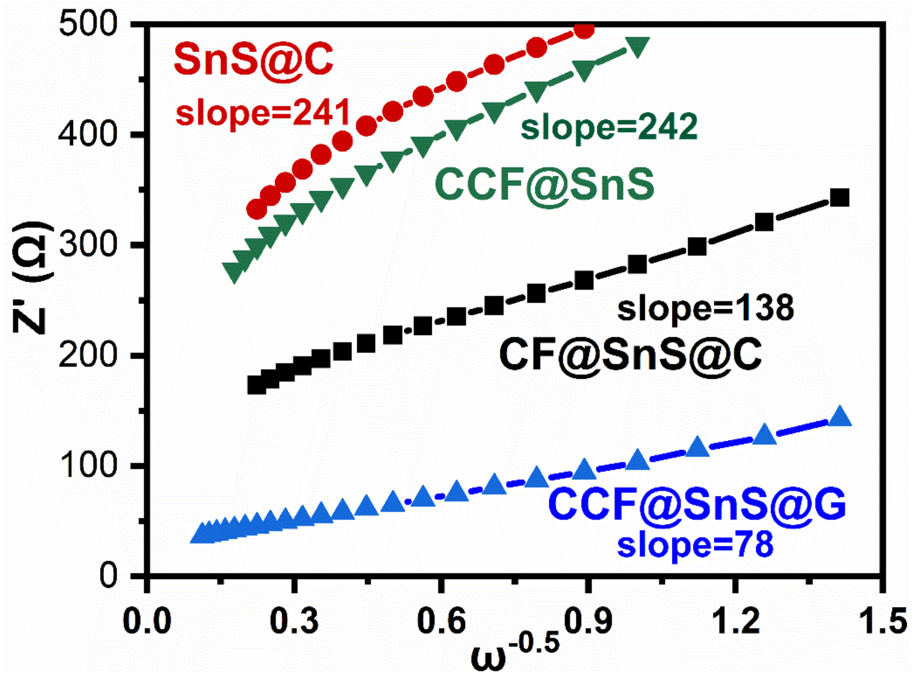

Figure S17 The relationship between Z' and $\omega^{-0.5}$ of different electrodes.

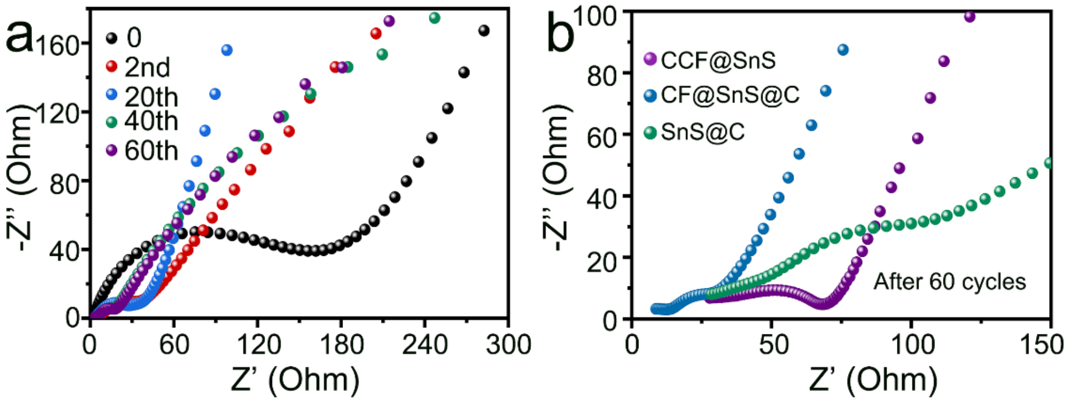

Figure S18 Nyqiust plots of CCF@SnS@G during cycles and the comparison for three electrodes of CCF@SnS, CF@SnS@C and SnS@C after 60 cycle.

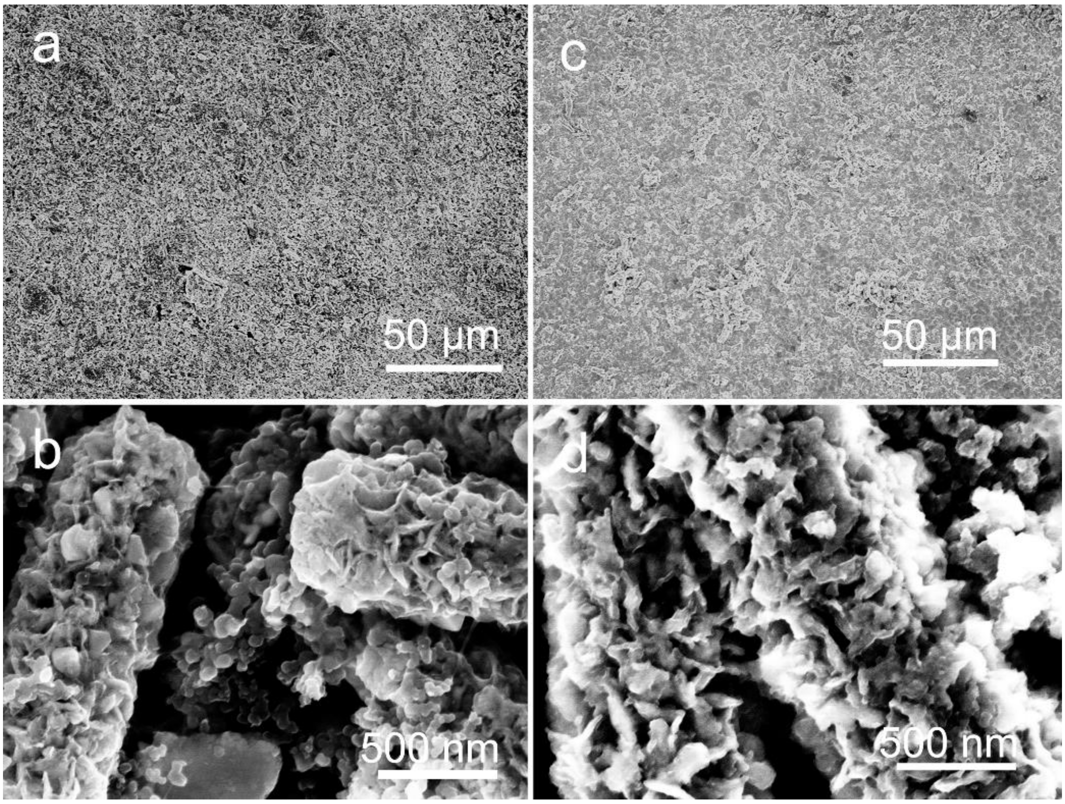

Figure S19 SEM images of the CCF@SnS@G electrode before (a,b) and after (c,d) cycles. The electrode displays integrated surface morphology without cracks and maintains original hierarchical structures after cycles, demonstrating excellent stability.

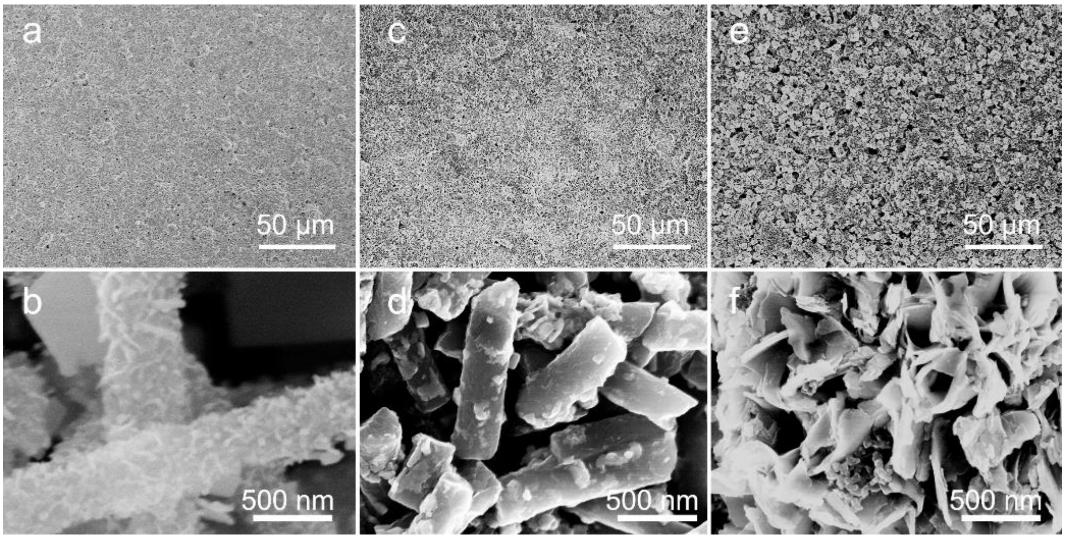

Figure S20 SEM images of the electrodes before cycles (a,b) CCF@SnS. (c,d) CF@SnS@C and (e,f) SnS@C.

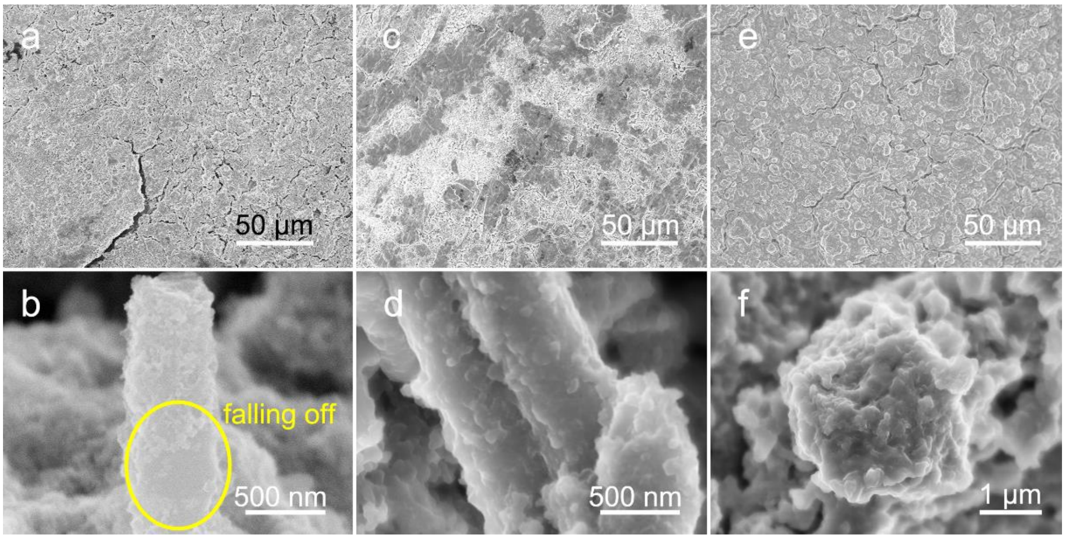

Figure S21 SEM images of the electrodes after cycles (a,b) CCF@SnS. (c,d) CF@SnS@C and (e,f) SnS@C. Compared with the SEM images before cycles in Figure S20, all of the three electrodes exhibit obvious changes in morphology and structures. Different levels of cracks on the electrode surface could be observed from the low magnification images and the original structures are also destroyed. The serious breakages and shed of active materials in

CCF@SnS are noticeable, while the hierarchical structures of CF@SnS@C and SnS@C are covered by thick SEI film.

Table S2 The electrochemical performance of SnS-based materials in SIBs

Materials	High rate	Cycle performance	Reference
SnS@C-rGO	336 mAh g^{-1}@3.2 A g^{-1}	524 mAh g^{-1}@0.1 A g^{-1}, 100 cycles	1
3D porous interconnected SnS/C	145 mAh g^{-1}@10 A g^{-1}	266 mAh g^{-1}@1 A g^{-1}, 300 cycles	2
SnS/C nanofiber	230 mAh g^{-1}@2 A g^{-1}	349 mAh g^{-1}@0.2 A g^{-1}, 1000 cycles	3
GF-SnS network	400 mAh g^{-1}@30 A g^{-1}	1010 mAh g^{-1}@0.1 A g^{-1}, 200 cycles	10
Graphene Survives SnS Bundles	345 mAh g^{-1}@20 A g^{-1}	~650 mAh g^{-1}@3 A g^{-1}, 1600 cycles	11
SnS@SNCF-55	289 mAh g^{-1}@3.2 A g^{-1}	332 mAh g^{-1}@1 A g^{-1}, 500 cycles	12
SnS@graphene hybrid architecture	308 mAh g^{-1}@7.3 A g^{-1}	308 mAh g^{-1}@0.1 A g^{-1}, 250 cycles	13
SnS/3DNG	404.8 mAh g^{-1}@6 A g^{-1}	509.9 mAh g^{-1}@2 A g^{-1}, 1000 cycles	14
C/SnS@C	120 mAh g^{-1}@10 A g^{-1}	240 mAh g^{-1}@1 A g^{-1}, 2000 cycles	15
N-S co-doped C@SnS/graphene	287.6 mAh g^{-1}@3.2 A g^{-1}	496.1 mAh g^{-1}@0.1 A g^{-1}, 70 cycles	16
CCF@SnS@G	433 mAh g^{-1}@10 A g^{-1}	541.4 mAh g^{-1}@2 A g^{-1}, 100 cycles	**This work**

Reference

[1] S. Zhang, G. Wang, Z. Zhang, B. Wang, J. Bai, H. Wang, *Small* **2019**, *15*, 1900565.

[2] C. Zhu, P. Kopold, W. Li, P. A. Aken, J. Maier, Y. Yu, *Adv. Sci.* **2015**, *2*, 1500200.

[3] J. Xia, L. Liu, S. Jamila, J. Xie, H. Yan, Y. Yuan, Y. Zhang, S. Nie, J. Pan, X. Wang, G. Cao, *Energy Storage Materials* **2019**, *17*, 1-11.

[4] D. H. Youn, S. K. Stauffer, P. Xiao, H. Park, Y. Nam, A. Dolocan, G. Henkelman, A. Heller, C. B. Mullins, *ACS Nano* **2016**, *10*, 10778-10788.

[5] Z. Deng, H. Jiang, Y. Hu, C. Li, Y. Liu, H. Liu, *AIChE J.* **2018**, *64*, 1965-1974.

[6] S. Mei, W. An, J. Fu, W. Guo, X. Feng, X. Li, B. Gao, X. Zhang, K. Huo, P. K. Chu, *Electrochim. Acta* **2020**, *331*,135292.

[7] L. Yao, M. Nie, C. Zhu, R. Cai, W. Xia, L. Sun, F. Xu, *Electrochim. Acta* **2019**, *297*, 46-54.

[8] J. Ai, Y. Lei, S. Yang, C. Lai, Q. Xu, *Chem. Eng. J.* **2019**, *357*, 150-158.

[9] P. Xue, N. Wang, Y. Wang, Y. Zhang, Y. Liu, B. Tang, Z. Bai, S. Dou, *Carbon* **2018**, *134*, 222-231.

[10] D. Chao, C. Zhu , P. Yang, X. Xia, J. Liu, J. Wang, X. Fan, S. V. Savilov, J. Lin, H. Jin, Fan, Z. X. Shen, *Nat. Commn.* **2016**, *7*, 12122.

[11] D. Chao, B. Ouyang, P. Liang, T. T. T. Huong, G. Jia, H. Huang, X. Xia, R. S. Rawat, H. J. Fan, *Adv. Mater.* **2018**, *30*, 1804833.

[12] Y. Wang, Y. Zhang, J. Shi, X. Kong, X. Cao, S. Liang, G. Cao, A. Pan, *Energy Storage Materials*, **2019**, *18*, 366-374.

[13] T. Zhou, W. K. Pang, C. Zhang, J. Yang, Z. Chen, H. K. Liu, Z. Guo, *ACS Nano*, **2014**, *8*, 8323-8333.

[14] X. Xiong, C. Yang, G. Wang, Y. Lin, X. Ou, J. H. Wang, B. Zhao, M. Liu, Z. Lin, K. Huang, *Energy Environ. Sci.* **2017**, *10*, 1757-1763.

[15] S. Chen, K. Xing, J. Wen, M. Wen, Q. Wu, Yi Cui, *J. Mater. Chem. A* **2018**, *6*, 7631-7638.

[16] J. Shi, Y. Wang, Q. Su, F. Cheng, X. Kong, J. Lin, T. Zhu, S. Liang, A. Pan, *Chem. Eng. J.* **2018**, *353*, 606-614.
